# Vision-based tactile sensing enhanced by microstructures and lightweight convolutional neural network

**DOI:** 10.1038/s41378-026-01355-5

**Published:** 2026-06-15

**Authors:** Mayue Shi, Yongqi Zhang, Xiaotong Guo, Eric M. Yeatman

**Affiliations:** 1 Department of Electrical and Electronic Engineering, Imperial College London, London, UK; 2 College of Science and Engineering, University of Glasgow, Glasgow, UK

**Keywords:** Electrical and electronic engineering, Engineering, Materials science

## Abstract

Tactile sensing can provide a critical function in advanced interactive systems by emulating the human sense of touch to detect stimuli. Vision-based tactile sensors are promising for providing multimodal capabilities and high robustness, yet existing technologies still have limitations in sensitivity, spatial resolution and the high computational demands of deep learning-based image processing. This paper presents a comprehensive approach combining a novel microstructure-based sensor design and efficient image processing, demonstrating that carefully engineered microstructures can significantly enhance performance while reducing computational load. Without traditional tracking markers, our sensor incorporates a surface with micromachined trenches, as an example of microstructures which can modulate light transmission and amplify the visual response to applied force. The amplified image features can be extracted by an ultra-lightweight convolutional neural network to accurately infer contact location, displacement, and applied force with high precision. Through theoretical analysis, we demonstrate that the micro trenches significantly amplify the visual effects of surface deformation. Using only a commercial webcam, the sensor system effectively detected forces below 5 mN and achieved a millimetre-level single-point spatial resolution. Using a model with only one convolutional layer, a mean absolute error below 0.05 mm was achieved. The compliant sensor body and optical readout design make the system inherently compatible with soft robotic integration and immune to electrical crosstalk or electromagnetic interference that often affects electronic tactile arrays. These characteristics highlight its potential for reliable operation in complex human–machine environments.

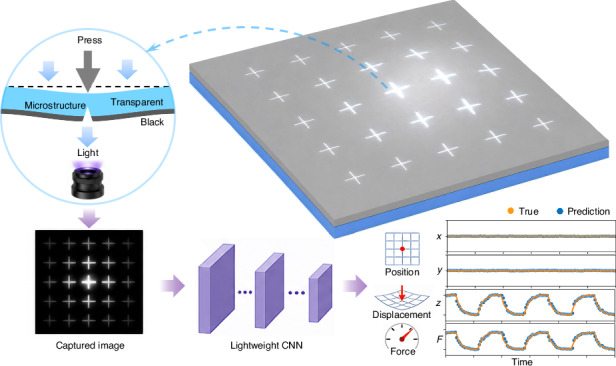

## Introduction

Soft tactile sensing is a key aspect of humanoid robots and other intelligent interactive systems^1^. It mimics the human sense of touch, enabling the detection of stimuli such as pressure, vibration and temperature^2–4^. Many applications have been developed in various fields^3^, such as robotics^5–7^, healthcare monitoring^8,9^, and prosthetics^10,11^. However, current soft tactile sensing technologies encounter significant challenges, especially in sensitivity, resolution and durability. Additionally, for large-scale sensing, which is critical for robots and prosthetics applications, current sophisticated sensing systems based on sensor arrays often have to face dramatic increases in complexity, electrical crosstalk and interference, and usually unacceptable cost. It is necessary to develop innovative solutions to enhance tactile sensing capabilities.

Vision-based tactile sensors (VBTSs), which utilise visual information to infer multimodal tactile properties, are promising to address the limitations in current soft tactile sensing. They can effectively detect location and magnitude of pressure, characterise surface textures, and operate in a wide range of scenarios. This innovative approach has been investigated for robotic perception^1^, offering rich information, high robustness and adaptability, low cost, and multimodal sensing capability^12^. Unlike conventional capacitive or piezoresistive tactile sensors, which rely on embedded electrodes and conductive traces that are prone to mechanical fatigue and electrical crosstalk, VBTS uses an optically readable elastomer layer and separate imaging components. This design reduces direct mechanical wear of electronic components and eliminates electrical interference between sensing elements, leading to improved durability and stability in long-term operation^13,14^. Furthermore, VBTS avoids the need for complex wiring and multiplexing circuits on soft sensor film, simplifying integration for large-area tactile sensing. Moreover, VBTSs are compatible with many computer vision methods, such as convolutional neural networks^2^. Recently, the rapid development of artificial intelligence technology has effectively boosted the development of VBTSs^15^.

The structure of a VBTS typically contains a sensor body and camera, among which the design of the sensor body is critical for the improvement of perception performance, while the camera module remains relatively standardised. The optimisation design of the sensor body generally involves several aspects, including novel structures, advanced materials and fabrication processes. With the rapid improvement of hardware designs, the last decade has witnessed the development of the VBTS towards miniaturisation, high performance and multimodality^16^. Current VBTSs are mainly based on two principles: displacement of markers with deformation of the sensor^17–22^, and irregular refraction, reflection and interference of light induced by surface deformation^13,23–28^, such as photometric stereo approaches.

Among marker-based sensors, GelForce is a well-known design for measuring the surface traction field on a robotic hand^20,21^. It has two layers of spherical markers embedded within a transparent silicone rubber sensor body. In addition, the TacTip family realises super-resolution tactile sensing for localisation tasks with a 3D-printed sensor body with markers, utilising pins on the sensor body to mimic the function of intermediate ridges within the human fingertip^18^. In contrast, photometric stereo-based sensors do not require markers. Early GelSight sensors captured high-resolution geometry and utilised photometric stereo to reconstruct the depth map and infer local force^25,29^. Later versions of GelSight introduced markers in the reflective membrane to enhance the capability to measure force and torque loads^24,30^. In addition, the Insight sensor^12^ presented a thumb-sized, conical elastomer-based VBTS with an embedded monocular camera. By combining photometric stereo and structured light techniques, it enables directional 3D force distribution sensing across the entire contact surface, achieving a spatial resolution of 0.4 mm and a force accuracy of approximately 0.03 N. Building upon this design, Minsight^13^ further miniaturises the sensor into the size of a human fingertip, capable of outputting real-time 3D force vector maps at 60 Hz.

In addition to sensor body design, improvements in lighting and system integration have further driven VBTS development toward miniaturised and simplified designs. For instance, WSTac^31^ introduced a whisker-inspired, self-illuminating VBTS that uses a mechanoluminescent elastomer to eliminate the need for external LEDs or light shielding modules, thus simplifying system design. Meanwhile, ThinTact^32^ proposed a lensless VBTS with a sensing field of over 200 mm^2^ using mask-based lensless imaging and a compact structure less than 10 mm thick.

Advances in optical systems and image processing have further enhanced VBTS performance. Multi-camera configurations have been proposed to address the limited field of view in single-camera setups^33^. Event-based cameras have also been introduced to boost temporal resolution and capture dynamic tactile information at kilohertz-level sampling rates, using asynchronous brightness changes as a stream of events. Evetac is an event-based optical tactile sensor designed to detect vibrations up to 498 Hz, leading to more adaptive and precise robotic manipulation^34^. A transfer learning algorithm was also explored to reconstruct normal force distribution and facilitate knowledge transfer across different sensor gels^35^.

Despite the progress in VBTS design, it is challenging for current solutions to balance high force sensitivity, spatial resolution, and computational efficiency. Many state-of-the-art systems require complex optical setups or deep neural networks with millions of parameters, making them unsuitable for real-time control in compact edge devices. In addition, neurophysiological studies of the glabrous skin of the human hand have shown that the median mechanical force detection thresholds of slowly adapting (SA I and SA II) receptors are approximately 1.3 mN and 7.5 mN^36^. Reaching these biological benchmarks remains challenging for artificial tactile sensors; however, they are critical for effectively mimicking human touch and enabling advanced tactile applications. A number of existing platforms have addressed capabilities such as shear-force sensing and multi-contact force distribution^12,21,24^, however, it is still challenging to achieve high sensitivity to out-of-plane deformation without complex illumination schemes or deep inference model architectures. The scenarios in which VBTS technology provides unique value are those involving surface deformation under contact load, making the underlying sensing problem inherently three-dimensional: the challenge is to infer the full contact state, such as location, depth, and force, from the two-dimensional optical projection of a deforming elastomer surface.

To address this gap, we propose Micro-VBTS, a vision-based tactile sensing system that integrates microstructure-enhanced optical modulation with a lightweight CNN model, achieving millinewton-level force resolution and millimetre-scale localisation while significantly reducing computational cost. The sensitivity of our system, with a detection limit approaching the natural tactile limits of the human fingertip, is promising to enable applications such as artificial electronic skin, humanoid robots, where precise force sensing is essential for delicate object manipulation and robust human–robot interactions.

**Fig. 1:**
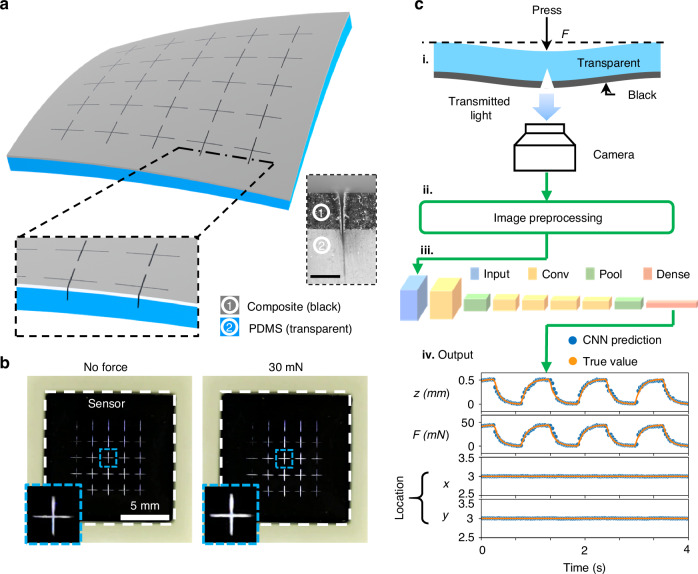
Structural schematic diagram and system design. **a** Structural design of the sensor body, and a sectional view showing trench structures in the film (scale bar: 200 μm). **b** Optical images of the sensor under different applied forces. Left: no force is applied. Right: a 30 mN force is applied at the circled cross centre in row 3, column 3. Diameter of contact area: 1 mm. **c** System design. i. Sensor body with micro trench patterns modulates light under pressure. ii. Camera captures images with transmitted light patterns and then iii. the images are pre-processed, and iv. feed into a lightweight CNN for analysis. v. Four outputs of CNN represent vertical displacement, applied force, and the location of the contact point on the sensor surface

We utilised microfabrication techniques to achieve high-precision structuring of soft composites, thereby enhancing the sensor’s performance. Although some studies have explored innovative structural designs for VBTSs, the potential benefits of integrating microfabrication approaches into these sensors have not been investigated. The microfabrication methods have been previously well-established for both soft and rigid materials, and proven effective in enhancing sensor performance in various micro-electromechanical systems. In our previous work, we presented preliminary results on developing microstructure fabrication processes for the VBTSs and a brief demonstration of brightness variation under applied force^37^. Here, we substantially extend that initial study to realise an end-to-end intelligent tactile microsystem, integrating microstructured sensor hardware with a lightweight machine learning pipeline for simultaneous inference of contact location, displacement, and force. (Fig. 1). We systematically calibrated and evaluated the system with different depths of CNN models for multi-axis inference, and provided a theoretical mechanics model to explain the behaviour of the microstructured sensor body. More importantly, this work suggests a broader design principle for intelligent tactile microsystems: co-design between sensor hardware and AI can substantially improve sensing and inference efficiency at the system level. With the proposed architecture, we achieve millinewton-level force resolution and millimetre-scale localisation, and further improve robustness through data augmentation techniques.

## Results

### Structural design and working principle

**Fig. 2:**
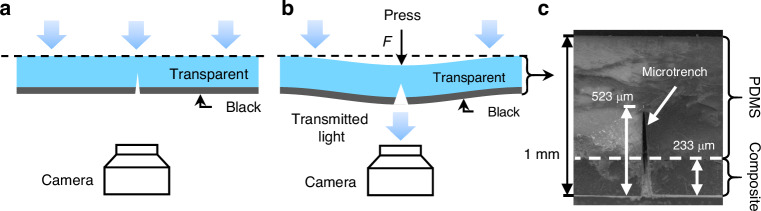
Principle of structure-enhanced vision-based sensor. **a** No applied force; **b** with applied force. The deformation allows light to transmit, forming specific patterns captured by the camera. **c** Scanning electron micrograph of a trench

As shown in Fig. 1a, the stretchable sensor body was composed of a transparent polydimethylsiloxane (PDMS) layer and a thin black graphite/PDMS composite layer. A grid of 5 × 5 cross-shaped trench structures was precisely fabricated using ultraviolet laser cutting, with a depth of around halfway through the sensor body film (Figs. 1a and 2c). The distance between adjacent cross centres is 2 mm, with each cross measuring 1.5 mm in both length and width. The sensor body is clamped using a 3D-printed frame with a 1.6 × 1.6 cm square window. The data processing pipeline of the Micro-VBTS system, as depicted in Fig. 1c, involves image acquisition and preprocessing, followed by lightweight CNN processing to learn the patterns and features necessary for the prediction of displacement in depth (*z*-coordinate), applied force and location coordinates (*x*, *y*). As shown in the figure, the comparison between true values and CNN predictions demonstrates the system’s high accuracy.

Figure 2 illustrates the working principle of this Micro-VBTS sensor that leverages the deformation of microstructures to measure applied force. Initially, the laser-written trenches are nearly closed, allowing minimal light through the structure. When force is applied on the transparent side, the trench in the black layer opens, creating a clear visual feature even for very slight deformation of the overall diaphragm shape. Importantly, the optical effect is amplified by the high depth-to-width ratio of the trench. The detailed structure of a micro trench is illustrated in Fig. 2c. Images are captured by a camera under the sensor body. For instance, the images of our sensor body clearly showed distinguishable features under an applied force of only 30 mN (Fig. 1b), which indicates its high sensitivity. With the diaphragm structure and elastic sensor body, the sensor recovers spontaneously after removal of the applied force.

In this work, we focused on a cross-shaped microstructure design, which was designed to achieve efficient light modulation and enhanced deformation amplification under applied forces. Other microstructure geometries may also influence the sensing performance by altering the way light is scattered and how local stress distributions are generated. For instance, circular or square trenches, as well as hemispherical, conical, or pyramid arrays, could provide different deformation responses and image features, potentially improving spatial resolution and force sensitivity. Moreover, the choice of material, with variations in elasticity, transparency, and refractive index, could further affect both the mechanical response and optical contrast, thereby impacting the robustness and accuracy of force inference. These factors represent promising avenues for future exploration to optimise sensor performance for specific applications.

### Theoretical modelling and simulation

**Fig. 3:**
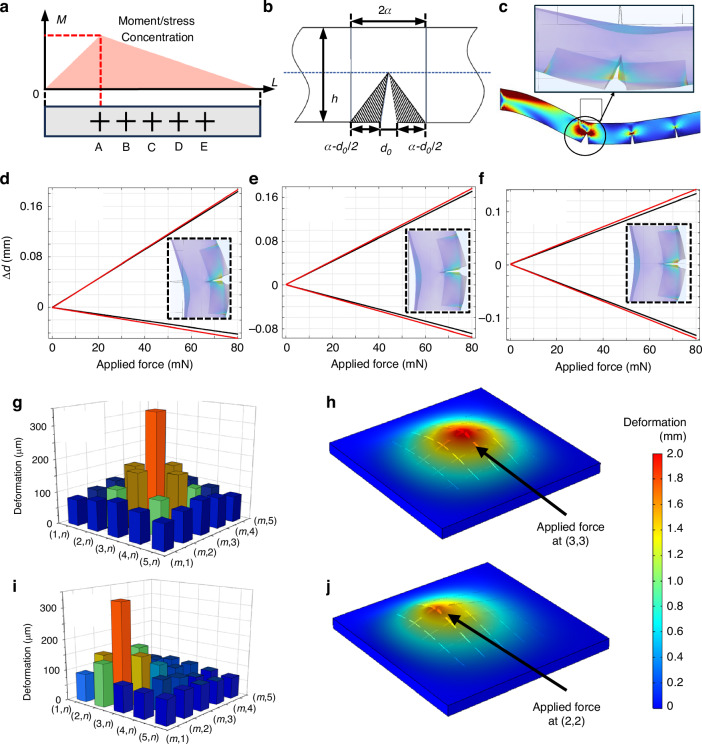
Modelling and simulation analysis. **a**, **b** Modelling of the sensor body structure. **a** Provides the illustration of simplified stress concentration. **c** Local stress distribution at location A under applied force based on FEA analysis. The deformation pattern of the notched beam at locations **d** A, **e** B, and **f** C simulated using COMSOL Multiphysics. The load range analysed was from 0 to 80 mN. Comparison between the simulated (black) and modelled (red) deformations demonstrates our model consistently predicts a slightly larger deformation at both edges, with discrepancies within 13% across varying applied loads. The average diagonal length (**g**, **i**) and deformation (**h**, **j**) of all 25 micro trenches under an applied force of 60 mN at location (3, 3) and location (2, 2)

We developed a comprehensive mathematical model to analyse the bending behaviour of a thin PDMS film with fixed boundaries and uniformly distributed V-shaped notches (Fig. 3 and Supplementary Note 1). With this model, we derived the extension of trench width (Δ*d*) under an applied force, which is critical for modulating light transmission and amplifying the variation. This model simplifies the complex behaviour of the sensor body film by representing it as an array of fixed-fixed beams. Each beam bends independently in response to an applied load, assuming that cross-sections remain undeformed during bending. A detailed modelling process is presented in the Supplementary Note 1.

As a result, for location $i(i\in \left\{A,B,C,D,E\right\})$, the deformations of the notch $\Delta {d}_{i}$ (Fig. 3b) can be expressed as:

1 $$\Delta {d}_{i}=\frac{3{M}_{i}}{{d}_{0}E\alpha }$$

Here, ${M}_{i}$ is the bending moment at location *i*; ${d}_{0}$ denotes the original trench width without applied force; *E* denotes the effective Young’s Modulus of the film, determined through experimental calibration; $\alpha$ is the geometric depth of the trench, also approximately half of the entire film thickness ($h\approx 2\alpha$) in our sensor.

To validate the mathematical model, we compared the finite element analysis (FEA) results with the analytical predictions (Fig. 3). Using COMSOL Multiphysics, we performed simulations over a range of applied loads from 0 to 80 mN, observing deformation patterns that closely matched our theoretical predictions. The applied force range was selected to represent fine-force sensing scenarios and to match the following experimental testing conditions. Further FEA was conducted (Fig. S2) to model the deformation and stress distribution of the sensor body under a 60 mN force applied at the central location. The material properties in our simulation were modelled assuming linear elasticity. The analysis revealed significant deformation around the contact point, affecting the micro trench’s luminous flux. To investigate the change of microtrench width across the 5 × 5 array, simulations were conducted for two scenarios (Fig. 3g–j): a 60 mN force applied at the central location (3,3) and at an off-centre location (2,2). Similar to our previous study^37^, we use the average diagonal length of the quadrilateral at the cross centre to represent the deformation of the microtrench under an applied force. Figure 3g, i presents the average diagonal length at the 25 cross centres for two scenarios. Both results indicated significantly larger variation at the contact point compared to other cross centres, which results in amplified light transmission and enhanced visual features, benefiting further feature extraction.

Furthermore, the deformation results reveal that deformation patterns were radially symmetrical across the 5 × 5 array when the central location (3,3) is pressed (Fig. 3h). Locations closer to the point of contact experience greater deformation compared to other cross centres, but with less contrast compared to the average diagonal length results, shown in Fig. 3g. In Fig. 3j, where the off-centre location (2,2) is pressed, points such as (5,1), (4,4), and (5,5), which are farther from the contact point, experience less deformation compared to their counterparts when the central location (3,3) is pressed.

The simulation primarily focused on normal force application. To further demonstrate the sensor’s behaviour under more realistic contact conditions, we also included an off-axis loading scenario in which a 45° force was applied (Fig. S9). This introduces both normal and tangential force components to evaluate the microstructure’s response to shear stress and deformation. Furthermore, we conducted parametric COMSOL simulations (Figs. S6 and S7) to briefly analyse how geometric factors such as trench arm length and spacing influence local stress distributions and deformation.

Although in-plane motion and shear force tracking are not yet included, the current sensor design is theoretically extendable to multi-axis force detection through additional calibration. In fact, high-resolution tracking of lateral motion using the cross-shaped features as references is straightforward, and observing differential motion of these features would provide detailed shear information. We have not focused on this in the paper because these functions are well provided by 2D markers and may benefit less from the 3D micro-structuring. Fig. S9 shows asymmetric widening of cross arms and differential lateral motion across neighbouring trenches.These optical cues can be exploited by a vision pipeline for shear direction/magnitude estimation. These results support the potential for calibrated shear sensing in future work.

It should be noted that the 5 × 5 grid used in our experiments serves only as a calibration matrix and does not define the intrinsic spatial resolution of the sensor. Since the image patterns exhibit distinct variations even when the contact point lies between calibration crosses (Fig. S10), localisation accuracy can be further improved by adopting a denser calibration grid without modifying the physical structure. Moreover, increasing the density of microstructures or employing a higher-resolution camera could push the resolution limits further.

### Experimental set-up and data collection

The fabrication of the sensor body involves two key steps: the fabrication of the multi-layer elastomer film, followed by a high-precision patterning process using ultraviolet (UV) laser cutting. During the multi-layer film fabrication, spin coating was used to ensure uniformity and precise control over the film thickness. For the laser cutting process, a high-precision UV laser cutter was employed, achieving a high aspect ratio microtrench structure with an opening width of approximately 40 μm. Detailed fabrication procedures of the sensor body are provided in Fig. S4 and ‘Methods’^37^. According to scanning electron microscopy measurement (Fig. 1e), the total thickness of the sensor body is 1 mm, while the composite layer is ~230 μm. The depth of the micro trench was ~523 μm.

**Fig. 4:**
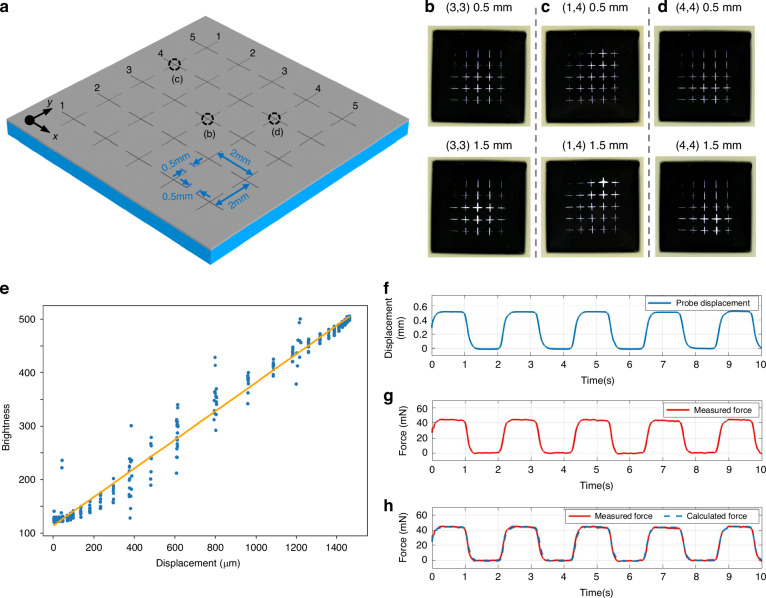
Sensor response to applied force. **a** Structure and parameters of the sensor body. **b**–**d** Optical images captured using a camera at three typical locations. The data above each image indicates the contact location and the displacement of the linear motor toward the sensor. For example, (3,3) indicates the applied force was at the cross centre in row 3, column 3. Diameter of contact area: 1 mm. **e** Brightness of the central region (3,3) and linear regression on the displacement–brightness data. **f**–**h** The force–displacement calibration of a testing point (3,3). **f** Displacement and **g** force were measured to calibrate the coefficient *k*_33_. **h** Comparison of the measured force and the calculated force according to the displacement over time (*k*_33_ = 85.4 mN/mm)

The performance of the Micro-VBTS was tested with a bespoke experimental setup, as shown in Fig. S5 and ‘Methods’. A commercial Logitech C922 camera (1920 × 1080 pixels resolution) was used to capture images at 30 frames per second (FPS) during force applications. We tested the sensor with a single contact point located at the centre of all crosses. Displacements of 0–0.5 mm, 0–1.0 mm, and 0–1.5 mm were applied against the sensor surface using a linear motor driving a cone-shaped probe (1 mm tip diameter) repeatedly at 1 Hz, with all 25 sensing points tested under consistent characterisation conditions and ambient lighting. The displacement of the linear motor was synchronously recorded. It is obvious that images captured under these conditions showed clear distinctions. The results from three typical testing points of (3,3), (1,4) and (4,4) are shown in Fig. 4b–d. The images indicate that crosses close to the contact point had high luminous flux and wide trenches, which is consistent with the modelling result. Additionally, the deformation of the film led to the displacement of crosses in images. This result was predicted by finite element simulation as well.

A quantitative image brightness-displacement analysis is shown in Figs. S3 and 4e. To investigate the sensing mechanism, we analysed the variation of optical brightness under controlled vertical displacement (*z*). A total of 300 video frames (30 FPS, 0–1.5 mm displacement range) were recorded while applying a vertical force at (3,3), the centre of the sensor surface. Each video frame was first cropped to a central area and then divided into 25 equally sized regions (60 × 60 pixels), forming a 5 × 5 grid. For each pixel, we extracted the value (*V*) channel from the HSV colour space, applied a threshold (*V* < 32) to suppress background noise, normalised the values to [0,1], and summed the V value of all pixels to quantify regional brightness.

Figure S3 shows the displacement–brightness scatter plots for all 25 regions. A clear monotonic trend is observed, particularly in the centre region where the force is applied. The central region (3,3) shows the steepest slope, indicating the strongest brightness modulation in response to vertical displacement. Peripheral regions exhibit smaller slope values due to less mechanical deformation.

To further quantify sensitivity, we performed linear regression on the displacement–brightness data of the central region (3,3). As shown in Fig. 4e, the slope of the fitted line reflects the local optical sensitivity to deformation. The linear fit yields the equation that $\mathrm{Brightness}=k\times z+b=0.2676z+113.45$, where *k* = 0.2676 (µm^−1^) denotes the optical sensitivity (i.e. change in brightness per unit displacement), and *b* = 113.45 corresponds to the baseline brightness when no force is applied. This quantification provides a clear metric for evaluating the microstructure-based brightness response to mechanical input.

This is especially beneficial for further in-depth image processing based on machine learning models to achieve high precision. Notably, while brightness is the primary measurable parameter used for sensitivity characterisation, the final machine learning model (such as CNN) can implicitly extract multiple image features beyond brightness. This enables the network to utilise global and local patterns across all 25 regions for robust prediction, rather than relying on a single high-sensitivity area.

The applied force (*F*) can be calculated based on vertical displacement (*z*-direction) based on a simple linear relationship. According to theoretical analysis and simulation^37^, the *F*-*z* relationship can be approximately expressed as $F\left(z\right)={k}_{{xy}}\times z$, where the coefficient *k*_*xy*_ represents the proportionality between vertical displacement and the applied force at location (*x*,*y*). To calibrate the coefficient *k*_*xy*_, we measured the applied force with a high-precision scale (MAXREFDES82, Analog Devices Inc.), as described in ‘Methods’. As an example, the displacement results from the point (3,3) are shown in Fig. 4f, g. According to the measured force and probe displacement, a linear curve fitting was performed, and the resulting *k*_33_ value was calibrated as 85.4 mN/mm. The coefficient of determination *R*^2^ was calculated as 0.99 at this point, which validated that the linear model is a good approximation of the force–displacement relationship, as shown in Fig. 4h.

### CNN model architecture and evaluation

**Fig. 5:**
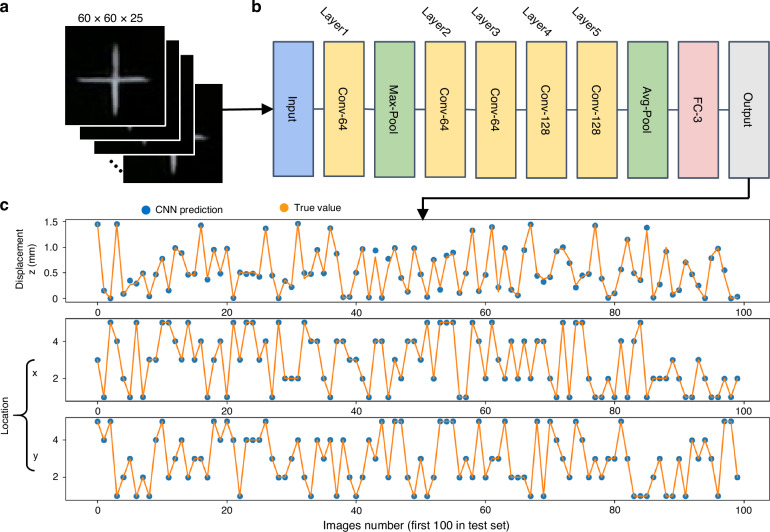
Architecture of the CNN model. **a** Pre-processed image as input. **b** Model architecture with 5 CNN layers (CNN_5). **c** A comparison between the predicted values by the CNN model and the true values for the first 100 images in the test set. *x* and *y* coordinates: contact position in units of inter-trench spacing (2 mm/unit); *z* coordinates: out-of-plane displacement (mm)

We have developed an ultra-light CNN model architecture to infer the location and magnitude of applied force, as shown in Fig. 5. The CNN model was selected because of its strong capability to extract spatial features from images, making it highly suitable for vision-based tactile sensing tasks. Lightweight architecture also enables faster inference and lower latency, which is significant for resource-constrained edge devices. Through hardware–model co-design, we optimised both the optical microstructure and the neural network architecture to complement each other: the microstructure amplifies subtle deformation-induced optical variations, while the CNN efficiently maps these enhanced visual cues to quantitative force and position outputs. Such co-optimisation between physical signal generation and computational inference ensures high sensitivity, robustness, and scalability for future embedded tactile systems.

The optical information collected by the camera was pre-processed before being passed to the CNN model (Fig. 1c). Specifically, the collected dataset included 225,000 frame images. We tested 25 points at cross centres on the sensor, using the linear motor to repeatedly apply three sequential displacement ranges (0.5 mm, 1 mm, 1.5 mm) starting from 0. For each displacement range, 3000 images were recorded, corresponding to 100 s at 30 FPS of data collection. After this, each image was processed by retaining only the value channel, and then cropped into 25 small images of 60 × 60 pixels, centred at the cross points. These cropped images were then input into CNN as 25 separate channels (Fig. 5a). To further enhance the diversity of the training data, data augmentation techniques were employed during the training process, including random translations within 1% of the image scale and rotations up to 1.8°. This approach helped to mitigate overfitting and improve the model’s robustness and generalisation capabilities. The training process was conducted on a high-performance computing cluster, allowing for efficient handling of the large dataset. Before splitting the dataset, it was shuffled to enhance randomness and prevent order bias. It was then divided into 70% for training, 15% for validation, and the remaining 15% for testing.

We tested models comprising only 1, 3, 5 and 7 convolutional layers. For instance, the model with 5 convolutional layers (Fig. 5b, denoted as CNN_5) has a total of 406k parameters. The image processing pipeline begins with preprocessing layers that rescale the pixel values. The image matrix values were rescaled to a range of 0 to 1 by dividing by 255, standardising the inputs and facilitating faster convergence. The convolutional layers progressively extract features, each followed by batch normalisation and ReLU activation to enhance learning efficiency and stability. The first convolutional layer outputs a shape of (54, 54, 64), while subsequent layers further refine the features, culminating in a (10, 10, 128) output after the fifth layer. The architecture also includes max pooling and average pooling layers to reduce spatial dimensions and computational complexity. Finally, the output is flattened and passed through a dense layer to predict the *x*, *y*, and *z* coordinates of the applied force.

Figure 5c demonstrates the prediction results of 100 sample images in the test set with our CNN_5 model, which reflects that the simple lightweight model performed well in predicting the applied forces’ *x*, *y*, and *z*-coordinates. Here, the *x* and *y* coordinates represent contact position in units of inter-trench spacing (1 unit = 2 mm), while the *z* coordinate represents out-of-plane displacement in mm. The predictions for the *x* and *y*-coordinates are particularly accurate. The displacement predictions are slightly less precise but still follow true values closely for most samples. These results indicate that the CNN model is effective for predicting the location and displacement. As demonstrated previously, the magnitude of applied force can be derived from the displacement using a simple linear relationship.

**Fig. 6:**
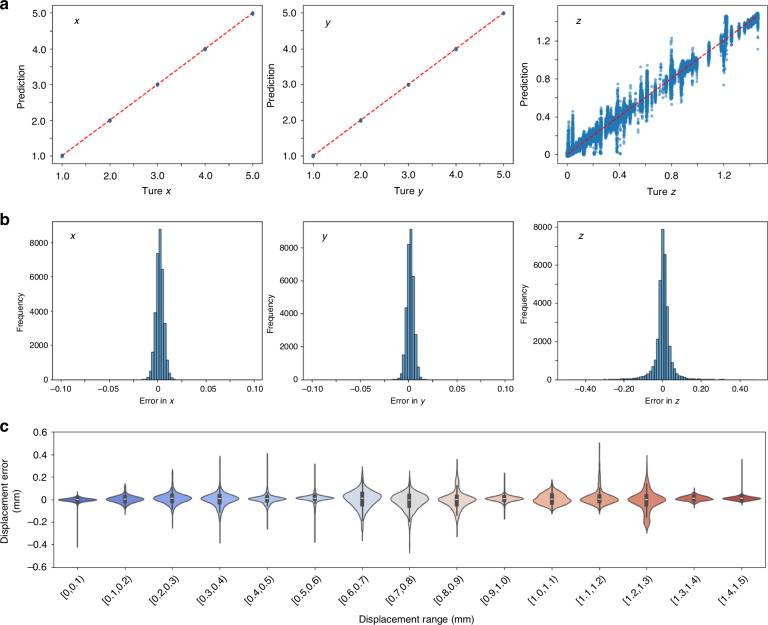
Statistical evaluation of model performance. **a** Scatter plots comparing the predicted values to the true values. **b** Histograms of the prediction errors. **c** Distribution of displacement prediction errors across different ranges of actual values

**Fig. 7:**
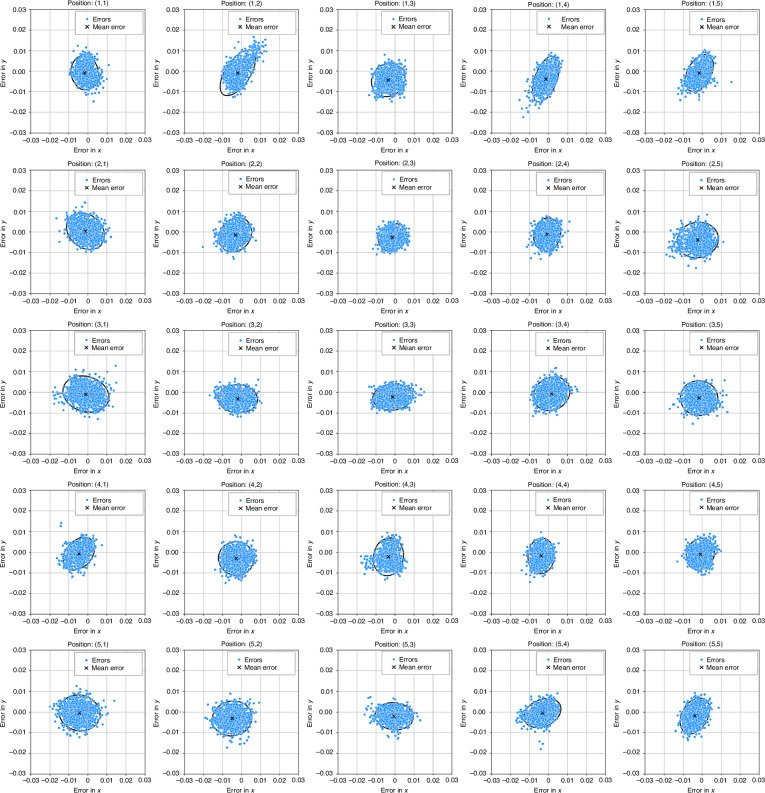
Error distribution for location resolution across different positions of the sensor

To systematically evaluate the model’s performance, we conducted a series of tests and statistical analysis using the test set, containing 15% of the images of the entire dataset. The results are shown in Figs. 6 and 7. In Fig. 6a, each subplot shows a clear correlation between the predicted and true values, indicated by the close alignment of the points along the red dashed line, which represents those predicted values equal to the true values. Consistent with the initial analysis of the results in Fig. 5c, the scatter plot for the *x*- and *y*- coordinates (left subplot) demonstrates an outstanding performance with most points falling on the line, indicating high accuracy in predicting the location of the applied force. For the *z*-direction or displacement (right subplot), there is more spread around the line, indicating higher variance in the predictions. However, the overall trend still shows a strong correlation between the predicted and true values. Notably, for the *x* and *y* directions in Fig. 6a, the five visible clusters correspond to five discrete testing positions; each cluster is related to a group of highly concentrated predictions, reflecting the robustness and precision of the CNN model.

Histograms of the errors (${r}_{i}$, where *i* represents the sample index) are shown in Fig. 6b. These errors, defined as as ${r}_{i}={y}_{i}-{\hat{y}}_{i}$, reflect the differences between the sensor outputs (${\hat{y}}_{i}$) and true values (${y}_{i}$). The residuals for the *x-* and *y-*coordinates (left and middle subplots) are narrowly distributed around zero, with the majority of residuals being very close to zero, confirming the model’s high accuracy in location prediction. The residuals for the *z-*coordinate (right subplot) have a wider distribution, indicating lower prediction accuracy. Nevertheless, 95% of the residuals fall within a narrow range, and the distribution peak is still centred around zero, suggesting that the predictions are unbiased.

To evaluate the sensor’s ability to measure displacement within different measurement ranges, we visualised the distribution of displacement prediction errors using a violin plot (Fig. 6c). This violin plot visualises the error distribution across different bins of actual values from 0 to 1.5 mm. Overall, these bins have a median error (white dot) near 0, indicating no significant systematic bias in predictions. Prediction errors concentrated around 0 in most bins, meaning that predictions align closely with actual values. The Micro-VBTS maintains high accuracy even for small displacements, with no evident performance degradation. Interestingly, for the smallest and largest bins, the direction of larger errors is notably asymmetric and occurs in opposite directions for these two bins. This phenomenon is understandable, as the model inherently learns to predict values within the range of sample data, which is within the 0–1.5 mm, rather than exceeding these limits. This also indirectly suggests that the model has effectively learned the characteristics of the sample data.

Furthermore, we analysed the *x*–*y* error distribution to assess location precision, as shown in the Fig. 7. This figure illustrates the *x*–*y* error patterns at 25 positions. In each subplot, the blue dots indicated individual error measurements, and the black error ellipses represented the 95% confidence interval. This figure shows that most distributions are concentrated around the origin, indicating relatively low errors. However, the spread of points in certain positions shows greater variability in error, suggesting slight localised differences in tactile sensing accuracy across the sensor.

In general, this model, containing 5 CNN layers, demonstrates excellent performance in predicting the location and displacement, particularly in location prediction. The slightly higher residual in the *z*-coordinate predictions suggests that there may be room for further optimisation of the model or the training process to improve accuracy in this dimension. Despite this, the CNN_5 model’s performance is robust and effective for precise tactile sensing.

**Fig. 8:**
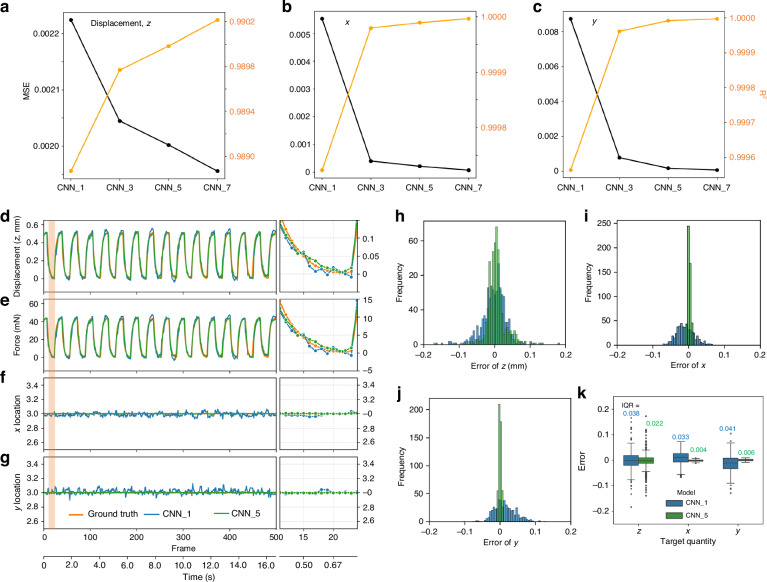
**Comparison of performance for different CNN models.** **a**–**c** Performance metrics for models with varying numbers of convolutional layers. **d**–**k** The experimental testing results and error analysis at location (3,3) using CNN_1 (blue) and CNN_5 (green). Specifically, ground truth and model predictions for **d** displacement and **e** calculated force in *z*-direction, **f** *x* and **g** *y*-coordinate results are shown. The shaded amber region highlights a low-force segment, with zoomed-in views provided on the right. **h**–**k** The error distributions and boxplots to evaluate prediction accuracy for this sensor system

To evaluate the impact of CNN depth on sensor performance, we systematically evaluated lightweight models with different numbers of convolutional layers, ranging from 1 to 7. Figure 8a–c presents the mean squared error (MSE) and *R*² values for different CNN models with varying numbers of layers (1, 3, 5, 7 layers). Table S1 shows a complete comparison of performance metrics for different models. The results indicate that increasing the number of CNN layers generally improves the model’s performance, as indicated by decreasing MSE and increasing *R*² values. While performance improves up to 5 layers for the *x* coordinate and continues to improve up to 7 layers for the *y* and *z* coordinates, there may be diminishing returns, particularly for the *x* and *y* coordinates. However, even the CNN_1 model with only 134k parameters achieved a relatively low MSE and an *R*² above 0.98. For all axes, a mean absolute error (MAE) below 0.05 mm was achieved, with specifically displacement (*z*) MAE to 0.03 mm. The model file saved in the Hierarchical Data Format (HDF5) is only 1.6MB, making it suited for deployment on memory- and power-constrained edge-computing devices.

Overall, our model achieved high precision. Due to the introduction of microstructures, the light flux variations caused by surface deformations on the sensor were amplified effectively. This amplification makes changes in graphical features more pronounced, allowing even shallow neural networks to effectively capture these features. Consequently, this reduces the computational and storage resource requirements, enabling efficient feature extraction with lightweight CNN architectures. As a result, it has the potential for deployment on edge devices, providing high-precision sensing while also reducing the computational burden on robots. This dual advantage highlights the efficacy of our design in balancing sensor performance with computational efficiency. Table S2 provides a comparative analysis of our results against other VBTS studies. The table highlights differences in optical systems, processing models, and errors in location and force/depth measurements.

To assess the sensor system’s capability in continuous multi-axis measurements at a specific location, we conducted tests at point (3,3) using models of varying depths (CNN_1 and CNN_5). Figure 8d–k shows the results of 500 consecutive frames captured at a rate of 30 FPS. The applied force was calculated with *k*_33_ = 85.4 mN/mm, as calibrated previously. The predicted displacement and force from both CNN_1 and CNN_5 aligned closely with the ground truth. This strong correlation validated the system’s high temporal accuracy and reliability during continuous testing. Both models achieved relatively accuracy, but CNN_5 yields visibly smoother and more stable predictions, as emphasised in the magnified zoomed-in views in Figure 8d-g. Notably, the zoomed-in segment corresponding to the low-force region clearly demonstrated that the Micro-VBTS system is capable of accurately capturing force, displacement, and positional changes even at low-force levels below 5 mN and displacements below 50 μm. These results were consistent with the comprehensive evaluation in Fig. 6c and Table S1. We have also included Supplementary Video S1, which provides a synchronised visualisation of the sensor’s response during force application. The video shows real-time changes in microstructure brightness and patterns, and corresponding output from the trained lightweight CNN_1 and CNN_5 models. The sensor demonstrated a validated displacement range of 0–1.5 mm, corresponding to an effective force range up to approximately 128 mN as derived from calibration results. The minimum detectable force, as shown in Fig. 8e, is below 5 mN. Notably, this sensitivity limit is comparable to the sensitivity thresholds of human mechanoreceptors^36^. These results highlight that the proposed Micro-VBTS system operates within a biologically relevant force range, enabling fine-force detection comparable to human touch. Furthermore, this range is potentially tunable by adjusting design parameters such as the thickness of the elastomeric film and the depth of the micro-trench structures.

While many VBTS systems struggle to sense deformation along the optical axis (z-direction) due to limited out-of-plane image contrast^38,39^, our system demonstrates robust *z*-axis performance enabled by the microstructure-enhanced light modulation. This structural advantage enhances *z*-direction sensitivity and contributes to more accurate vertical displacement estimation. It is worth noting, however, that the slightly larger inference error observed in the *z*-direction compared to *x*–*y* localisation (as seen in Fig. 8) is partly attributed to the testing methodology. Specifically, *x*–*y* data were collected at 25 discrete locations, allowing the model to learn distinct image features for each calibration point. In contrast, *z*-direction data were collected continuously using a linear motor, resulting in subtler and less distinguishable image variations. This increases the learning difficulty for the network.

The error histograms in panels (h)–(j) further quantify model accuracy. CNN_5 shows tighter and more symmetrical error distributions centred around zero for all axes, indicating improved consistency over CNN_1. Finally, Fig. 8k shows the boxplot for sensing errors for displacement, *x*, and *y* locations. The result reflected that the model CNN_5 consistently performs better than CNN_1, as indicated by its lower interquartile ranges.

Since brightness is used as a primary image feature in our model, we note that ambient lighting conditions could potentially affect sensor performance. However, the model primarily relies on relative brightness variations rather than absolute values, which helps mitigate this effect. All experiments were conducted under natural ambient lighting without additional shielding, and the system showed robust and consistent performance, indicating a degree of resilience to typical illumination changes.

As a system-level investigation, microstructure-enhanced optical modulation generates amplified image features in this study sufficient for high-performance tactile inference using a single-convolutional-layer model, without deep architectures or large parameter counts. This finding suggests that computational complexity and sensing accuracy were decoupled when the optical front end was appropriately engineered, laying a critical foundation for effective inference on resource-constrained micro-sensing systems at the edge. More broadly, this work suggests a design strategy for intelligent sensing microsystems: physical amplification at the hardware level directly reduces the complexity required of the inference model, enabling high-performance sensing with a significantly simplified computational pipeline.

## Conclusions

In this paper, we propose a high-performance vision-based tactile sensing paradigm that integrates microstructures and computer vision. Based on this paradigm, we designed and implemented a Micro-VBTS using microfabricated micro-trenches and lightweight CNNs, and systematically evaluated its performance. This innovative sensor design incorporates a black composite layer with 25 cross-shaped micro-trenches, modulating the light luminous flux based on the applied force. Camera-captured images of the sensor body serve as the input for the ultra-light CNN model architecture to accurately infer the location, displacement and magnitude of applied force. We evaluated the models with 1–7 convolutional layers and found that the models effectively extract relevant features from the input images, facilitating precise predictions. The model architecture balances computational efficiency with high accuracy, making it suitable for real-time applications in IoT and edge-computing. The Micro-VBTS achieved accurate tactile inference down to the millinewton level, with a displacement MAE of 0.03 mm and localisation MAE below 0.05 mm. Its compatibility with computer vision methods enhances its potential for integration with and further enhanced by advanced AI systems. Furthermore, its soft sensor body makes it highly promising for seamless integration into advanced soft robots and wearable electronics.

In future research, this tactile sensing paradigm can be further developed and expanded for broader applications. These cross-shaped micro trenches in this work have demonstrated that well-engineered microstructures can effectively enhance tactile sensing by efficient light modulation and enhanced deformation amplification under applied forces. Other microstructure geometries may also influence the sensing performance by altering the way light is scattered and how local stress distributions are generated. For instance, circular or square trenches, as well as conical or pyramid arrays, could provide different deformation responses and image features, potentially improving spatial resolution and force sensitivity (Fig. S8). Moreover, the choice of material with variations in elasticity, transparency, and refractive index could further affect both the mechanical response and optical contrast, thereby impacting the robustness and accuracy of force inference. Additionally, the present study characterised the intrinsic sensing mechanism and performance of Micro-VBTS under controlled single-contact conditions, prior to the additional complexity introduced by multi-contact loading. The existing sensor design holds potential for multi-point tactile sensing by collecting and training on multi-point datasets. Future research will extend to multi-point simultaneous contacts, quantifying performance under varied contact geometries and a denser calibration. Comprehensive evaluation of multi-axis force interactions is also important in future studies. Furthermore, although this work focuses on the fundamental performance characterisation of the proposed Micro-VBTS, we recognise the importance of validating its practical utility in robotic scenarios. In future work, we will work on integrating the sensor into a robotic manipulation platform for task-level validation, for which the sensing performance characterised here provides a solid basis.

By integrating innovative sensor architecture, advanced microfabrication, and lightweight machine learning, this study establishes a microstructure-enhanced microsystem framework for vision-based tactile sensing. Rather than treating hardware and algorithms as separate modules, we propose a co-design paradigm that jointly optimises sensor microstructures and the machine learning pipeline, engineering the sensor itself to generate physical features that are inherently more tractable for machine learning to process. By amplifying critical physical features directly at the device level, this strategy significantly reduces the reliance of downstream models on complex feature extraction and high computational cost. Beyond demonstrating improved tactile sensing performance, this work provides a new route toward integrated device–information–algorithm co-design for future intelligent sensing microsystems.

## Methods

### Device fabrication

We first fabricated a thin PDMS substrate film. The PDMS base and curing agent (Dow Corning, SYLGARD 184 silicone elastomer) were mixed in a ratio of 10:1 (w/w), after which the mixture was spin-coated on a plate before being transferred to an oven for curing at 70 °C for 2 h. To prepare the graphite/PDMS composite, 10 wt% graphite (Sigma-Aldrich, 282863) was fully mixed with uncured PDMS (base: curing agent = 10:1, w/w) to form a black composite, enhancing image contrast. Toluene was added to the mixture to assist in uniform dispersion and adjust its viscosity. The mixture was spin-coated on the transparent PDMS film (300 rpm, 60 s) to ensure uniformity. The film was transferred to an oven at 70 °C for 4 h. After preparing a double-layer elastomer film, the micro trenches were fabricated with high-precision ultraviolet laser cutting. The wavelength of the UV laser was 355 nm. As shown in Fig. S4b, the multi-layer film was first placed on a glass plate, after which the film was patterned with the cross-shaped trench structure using a computer-controlled laser cutter.

### Experimental set-up

The bespoke experimental setup aims to repeatedly apply vertical forces onto the sensor with a given displacement. The setup’s actuation mechanism consists of a magnetic linear motor (Faulhaber LM038004001) and a 3-D printed cone-shaped probe (1 mm tip diameter). The position sensor in the linear motor provides highly accurate displacement measurements with a precision of 1 µm and is capable of outputting and recording displacement data. The probe driven by the linear motor enables applying a controllable and high-precision displacement against the sensor surface.

### Calibration of the force–displacement relationship

We placed the targeted sensing point at the centre of the bespoke probe. A high-precision scale (MAXREFDES82) was placed beneath the VBTS, which measures the forces applied. Subsequently, the position of the probe was adjusted to a height where it just contacted the sensor body. This scale module integrates a precision strain gauge sensor and a 24-bit ADC, capable of measuring forces in the millinewton range with high resolution and stability. The sensor was connected to a laptop via USB for real-time data acquisition. It was calibrated using standard weights before use.

By initialising the linear motor with a pre-set displacement, we were able to measure the change of forces with the scale. We measured the force, and the sensing points located at the top left corner of the sensor were tested: (1,1), (2,1), (2,2), (3,1), (3,2), and (3,3). Hence, the rest sensing points can be estimated symmetrically. For each sensing point, displacements of 0.5, 1.0, 1.5, and 1.75 mm were applied. And each force application was repeated for 10 s with a frequency of 0.5 Hz.

## Supplementary information

Supplementary Information

Supplementary Video S1

## Data Availability

All data are available within the article or available from the corresponding author upon reasonable request.

## Code availability

The code used for machine learning is available via github.com/mayue-shi/MicroVBTS (10.5281/zenodo.20431024).
