# Supplementary material for: Vision-based tactile sensing enhanced by microstructures and lightweight convolutional neural network
Source: Microsyst Nanoeng. 2026 Jun 15;12:231. doi: 10.1038/s41378-026-01355-5 (PMC13265784; doi:10.1038/s41378-026-01355-5)
Supplement: Supplementary file 1 — Supplementary Information [file 41378_2026_1355_MOESM1_ESM.docx]

Supplementary Information

**Vision-Based Tactile Sensing Enhanced by Microstructures and Lightweight Convolutional Neural Network**

Mayue Shi^1^*, Yongqi Zhang^1^, Xiaotong Guo^1^ and Eric M. Yeatman^1^

^1^Department of Electrical and Electronic Engineering, Imperial College London, London, U.K.

^2^College of Science and Engineering, University of Glasgow, Glasgow, U.K.

*Email: m.shi16@imperial.ac.uk

Table of Contents

[Supplementary Note 1 | Mathematical Model and Analytical Approach (Figure S1.1-S1.16) 2](#_Toc211148510)

[References 15](#_Toc211148511)

[Supplementary Figure S2 16](#_Toc211148512)

[Supplementary Figure S3 17](#_Toc211148513)

[Supplementary Figure S4 18](#_Toc211148514)

[Supplementary Figure S5 19](#_Toc211148515)

[Supplementary Figure S6 20](#_Toc211148516)

[Supplementary Figure S7 21](#_Toc211148517)

[Supplementary Figure S8 22](#_Toc211148518)

[Supplementary Figure S9 23](#_Toc211148519)

[Supplementary Figure S10 24](#_Toc211148520)

[Supplementary Table S1 25](#_Toc211148521)

[Supplementary Table S2 26](#_Toc211148522)

Supplementary Note 1 | Mathematical Model and Analytical Approach (Figure S1.1-S1.16)

**1.1 FLEXIBILITY METHOD TO ANALYZE FIXED-FIXED BEAMS**

The flexibility method proves to be a valuable approach for analyzing statically indeterminate systems, enabling the elimination of redundant reactions within the system through successive replacements. In our specific case, we simplified the initial third-degree statically indeterminate system to a second-degree one by disregarding the axial indeterminacy and focusing on the shear and moment. Consequently, we can free one of the fixed ends, as shown in **Figure S1.1**, resulting in a degree of freedom (DOF) associated with translation (termed as DOF #1) and another associated with rotation (termed as DOF #2). This reduction in degrees of freedom streamlines the analysis and facilitates a more manageable understanding of the system's behavior.


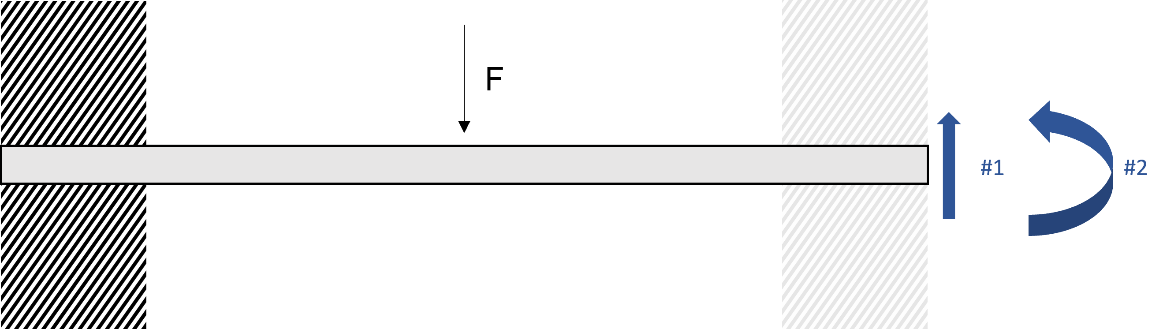


**Figure S1.1**: Using the flexibility method to analyze statically indeterminant systems. By releasing the boundary condition at one end (in this case, the right end), we are going two have two DOFs associated with translation (#1) and rotation (#2), respectively.

Given the nature of the fixed-fixed setup, the displacements in the translational direction, denoted as $\Delta_{1}$, and the rotational direction, denoted as $\Delta_{2}$, are constrained to be zero. It's important to note that we have established a convention where upward displacements are considered positive.

|  | $\Delta_{1}=\Delta_{2}=0$ | (S1) |
| --- | --- | --- |

Returning to the original system, comprising a fixed-fixed beam and a concentrated load F, we can deconstruct this system into the sum of the primary loading and the redundant reactions of a fixed-free beam, as illustrated in **Figure S1.2**.


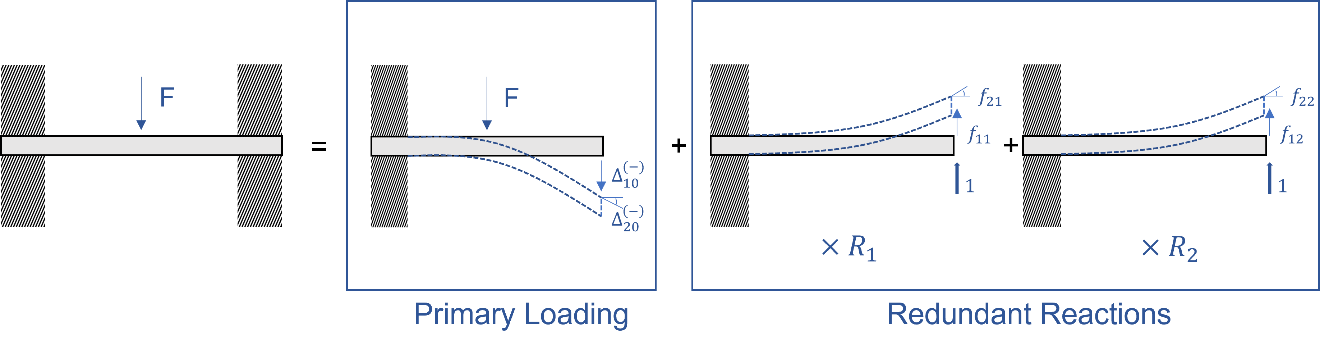


**Figure S1.2**: Full original system of a fixed-fixed beam could be dissected Primary Loading plus Redundant Reactions

The full original system of a fixed-fixed beam could be dissected as, 1. Primary loading: We disassemble the full system by removing the reaction at one end and allowing the structure to curve and displace accordingly. Translation will be denoted as $\Delta_{10}$, where the subscript 1 denotes the relevant degree of freedom (DOF #1), and 0 denotes the primary loading situation. The same logic applies to rotation, $\Delta_{20}$. In both cases, following our convention, $\Delta_{10}$ and $\Delta_{20}$ will be negative. 2. Redundant reactions: We reintroduce the redundant reactions by multiplying them by the actual yet unknown redundant forces $R_{1}$ and $R_{2}$. For practical purposes, we use a unit load of 1 for both redundant shear and moment to trigger responses denoted by flexibility coefficients $f_{11}$, $f_{12}$, and rotation coefficients $f_{21}$, $f_{22}$.

Based on where the load is applied, we are able to formulate the translations $\Delta_{10}$ and $\Delta_{20}$, as well as flexibility coefficients $f_{11}$, $f_{12}$, and rotation coefficients $f_{21}$, $f_{22}$ in terms of load $F$, length of the beam $L$, Young’s Modulus of the material $E$, and moment of inertia $I$. Then we can proceed to write out the compatibility equations to this system. For shear/translation $\Delta_{1}$, which ultimately is equal to 0, we have,

|  | $\Delta_{1}=\Delta_{10}+f_{11}R_{1}+f_{12}R_{2}=0$ | (S2) |
| --- | --- | --- |

Similarly, for rotation $\Delta_{2}$, we have,

|  | $\Delta_{2}=\Delta_{20}+f_{21}R_{1}+f_{22}R_{2}=0$ | (S3) |
| --- | --- | --- |

We can then put these relations in a matrix form as $\left\{ \Delta-\Delta_{0} \right\}=\left[ f \right]\left\{ R \right\}$ to solve for redundant reaction forces $R_{1}$ and $R_{2}$ via calculating the determinant of the flexibility matrix $\left[ f \right]$, and ultimately obtain the corresponding moment and shear plots.

**1.2 SAINT-VENANT’S PRINCIPLE IN FINITE ELEMENT METHOD**

Saint-Venant’s principle, a fundamental concept in structural engineering, is a commonly utilized notion among structural engineers. Essentially, Saint-Venant’s principle asserts that the precise distribution of a load becomes less critical as we move away from the loaded region, as long as the overall load resultants remain accurate. We leverage the Saint-Venant’s principle, with a specific emphasis on its application within the domain of finite element (FE) analysis.

We can begin with a relatively straightforward scenario: a thin rectangular plate featuring a circular hole at a certain distance from the loaded edge, subjected to axial pulling. The main question to explore is the significance of the actual load distribution when we are particularly interested in stress concentration around the hole. In this case, we consider three distinct load types applied at the rightmost boundary:

Scenario 1. Consistent axial stress of 100 MPa

Scenario 2. Symmetric parabolic stress distribution with peak amplitude 150 Mpa

Scenario 3. Centrally placed point load, engineered to yield an equivalent resultant compared to the previous two load cases

The stress distribution at the hole, as depicted in **Figure S1.3**, conspicuously showcases that the mode of load application doesn’t notably influence the stress concentration around the hole. The critical factor lies in the hole being sufficiently distant from the applied load.


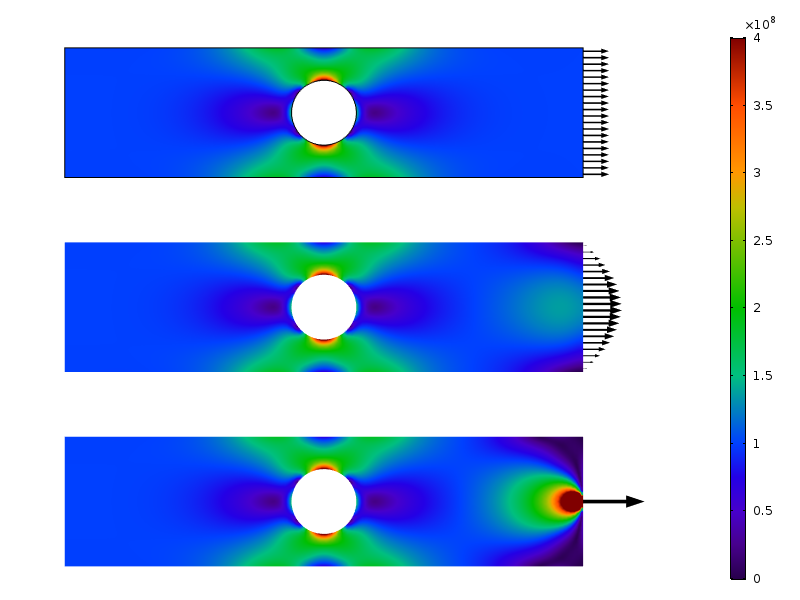


**Figure S1.3**: Von Mises stress contours for the three load cases. These contours visually demonstrate that the Von Mises stress distribution around the hole remains consistent regardless of the mode of load application. Scenario 1. Consistent axial stress of 100 Mpa, Scenario 2. Symmetric parabolic stress distribution with peak amplitude 150 Mpa, Scenario 3. Centrally placed point load, engineered to yield an equivalent resultant compared to the previous two load cases.

An alternative and insightful way to depict this scenario is through principal stress arrows. This visualization emphasizes the stress field as a flux, providing a clear representation of stress redistribution. **Figure S1.4** showcases the principal stress arrows for the three load cases. These arrows effectively illustrate stress redistribution and provide valuable insights into how stress is distributed in the structure for each load case.


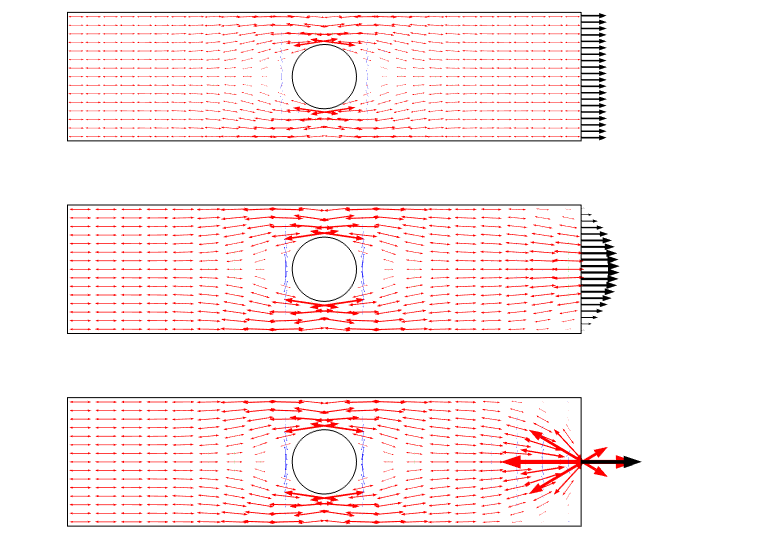


**Figure S1.4**: Principal stress plot for the three load cases (blue arrows demonstrate compression, while red arrows extension). Note a singularity when point load is used in Scenario 3.

Analyzing stress distribution along a line, as depicted in **Figure S1.5**, provides valuable insights. It becomes evident that as we move away from the loaded edge, approximately reaching the width of the plate, the stress profiles from the three cases start to align and converge. This convergence point serves as a critical observation, signifying that beyond this distance from the loaded edge, the stress distribution is no longer influenced by the distinct load types applied. Instead, it settles into a consistent pattern that remains largely unaffected by the specific way the load is applied.


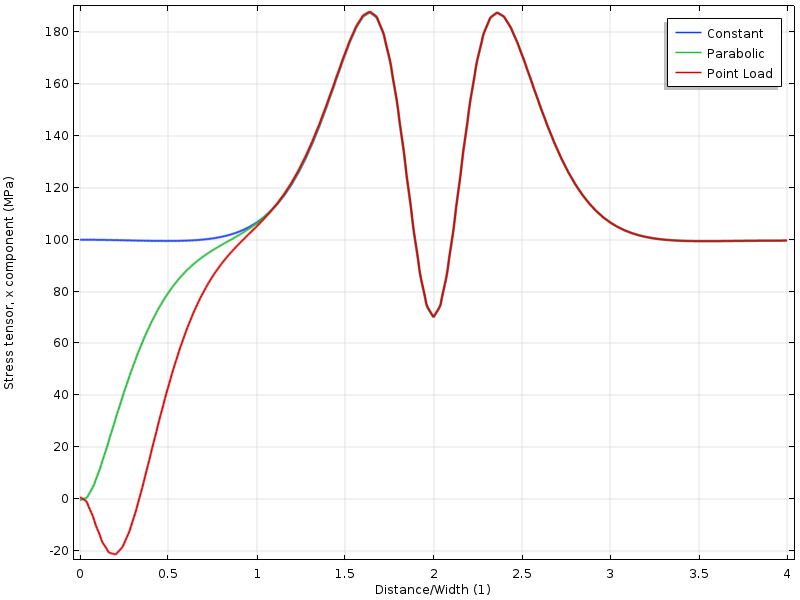


**Figure S1.5**: Stress along the upper edge, as a function of the distance from the loaded boundary. The distance is normalized by the width of the plate.

When relocating the hole closer to the loaded boundary, a noticeable transformation occurs in the stress distribution around the hole, signifying a dependency on the load distribution. Illustrated in **Figure S1.6**, the point where the stress fields from the three load cases come together is now at a distance twice as far from the loaded boundary compared to the previous scenario. This shift in the convergence distance holds significant implications—it emphasizes a fundamental principle: for Saint-Venant’s principle to hold, stresses must be allowed to redistribute freely. However, the presence of the hole in this case partially hampers this redistribution, leading to the observed alterations in stress patterns.


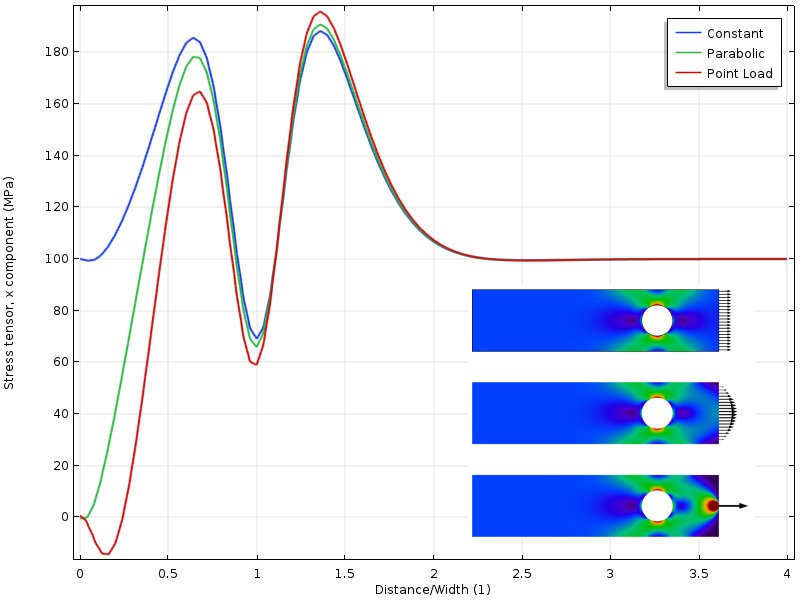


**Figure S1.6**: Stress along the upper edge with the hole closer to the loaded boundary. The distance to the point where the stress fields from the three load cases converge is now twice as far from the loaded boundary.

It's important to highlight that Saint-Venant’s principle emphasizes that there is negligible difference in the stress state at a distance comparable to the linear dimension of the loaded area. However, a key consideration is that the loaded area to be considered may not necessarily be the actual area that is physically loaded. When the hole is situated far away, we often determine stress concentration factors using handbooks. The handbook approach implicitly assumes even load distribution, similar to the first load case. Consequently, even if the actual load is applied to a small portion of the boundary, the critical distance is related to the size of the entire boundary.

In contrast, when employing the finite element method (FEM), the hole can be placed very close to the load. The limiting factor is the well-defined load distribution from a physical standpoint. However, it's crucial to acknowledge that assumptions about redistribution implicitly assume a specific load distribution, which may not align with the actual distribution. This distinction underscores the nuanced nature of stress analysis and the implications of assumptions on load distribution in different methodologies.

**1.3 DEFORMATION MODELING OF LOADING UNNOTCHED BEAM**

As introduced, upon simplification, the system transforms into a beam with two fixed ends, as illustrated in **Figure S1.7**. This configuration introduces three potential reactions at each end, rendering the system statically indeterminate to the third degree. It's noteworthy that one of these reactions operates exclusively in the axial direction and remains uncoupled from both shear and moment. To streamline the analysis practically, we can disregard this axial reaction, consequently simplifying the system to a second-degree static indeterminacy.


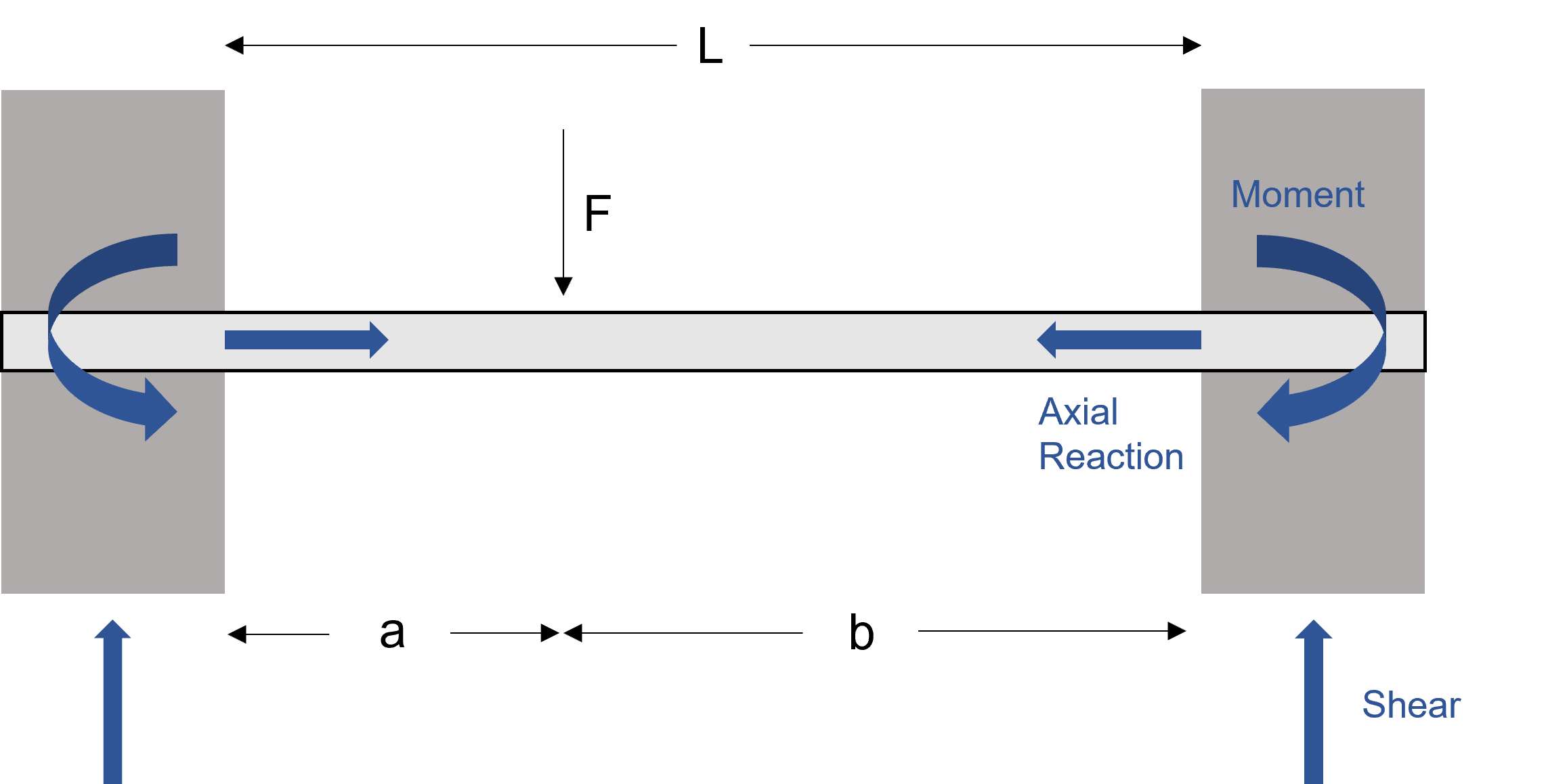


**Figure S1.7**: The reactions to bending of a generic fixed-fixed beam. In this illustration, a beam of length L is fixed at both ends, and a concentrated load F applied at an arbitrary location induces three reactions, namely axial, shear, and moment. However, for pragmatic considerations, we concentrate solely on analyzing the shear and moment reactions, as the axial reaction remains decoupled from these responses.

This simplification allows us to focus solely on shear and moment reactions for a more targeted analysis. Additionally, we proceed with the assumption that the beam exhibits linear elastic behavior, maintaining a uniform Young’s modulus (E) and moment of inertia (I) throughout. This assumption is fundamental to our analytical approach and provides a solid basis for modeling the beam's deformation patterns accurately.

The flexibility method serves as a powerful tool for analyzing statically indeterminate systems. This approach involves eliminating redundant reactions within the system and replacing them with appropriate degrees of freedom (DOFs), enabling a systematic analysis of the structure. By applying the flexibility method systematically, we can construct a detailed moment diagram illustrating the bending of a fixed-fixed beam. This comprehensive diagram accounts for the loads applied at notched locations labeled as A, B, C, D, and E, each specified in **Figure S1.8**.


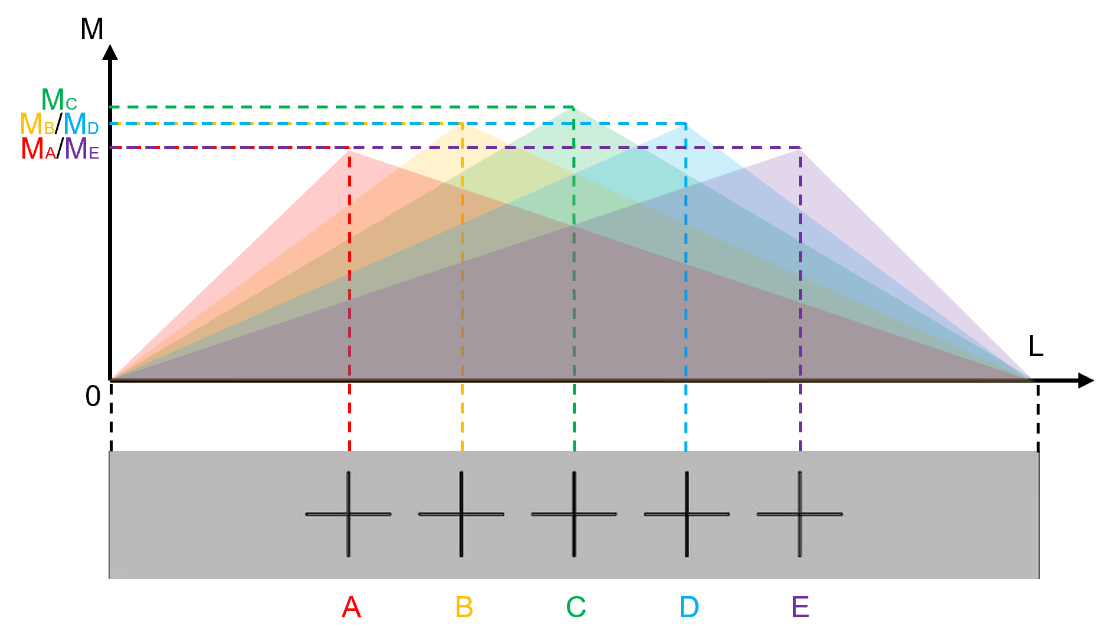


**Figure S1.8**: Comprehensive moment diagram of the system, taking into consideration the loads applied at notched locations A, B, C, D, and E. The moments for each location are denoted correspondingly as $M_{A}$, $M_{B}$, $M_{C}$, $M_{D}$, and $M_{E}$.

The maximum moment, $M_{max}$, at each location is calculated using $M_{max}=\frac{Fab}{L}$, where $F$ is the applied load, $L$ is the length of the beam, and $a$ and $b$ are distances from the point of application to the two ends of the beam. Notably, the maximum moment occurs at the central location $C$, where $M_{C}=\frac{FL}{4}$ due to the symmetric arrangement of notches. Moreover, owing to the symmetrical layout of the notches, we can observe that $M_{A}=M_{E}$ and $M_{B}=M_{D}$, further simplify the analysis and reduce the number of calculations needed.

We began the simplification of the thin film by conceptualizing it as an array of fixed-fixed beams. In this representation, during bending, the beams do not exert pressure on each other, assuming minimal deformation of their cross-sections. Therefore, when an external load is applied to a specific region of the surface, the film undergoes bending. This bending effect can be accurately understood by considering that each beam within the array responds independently, aligning with the distinct areas of the applied load.


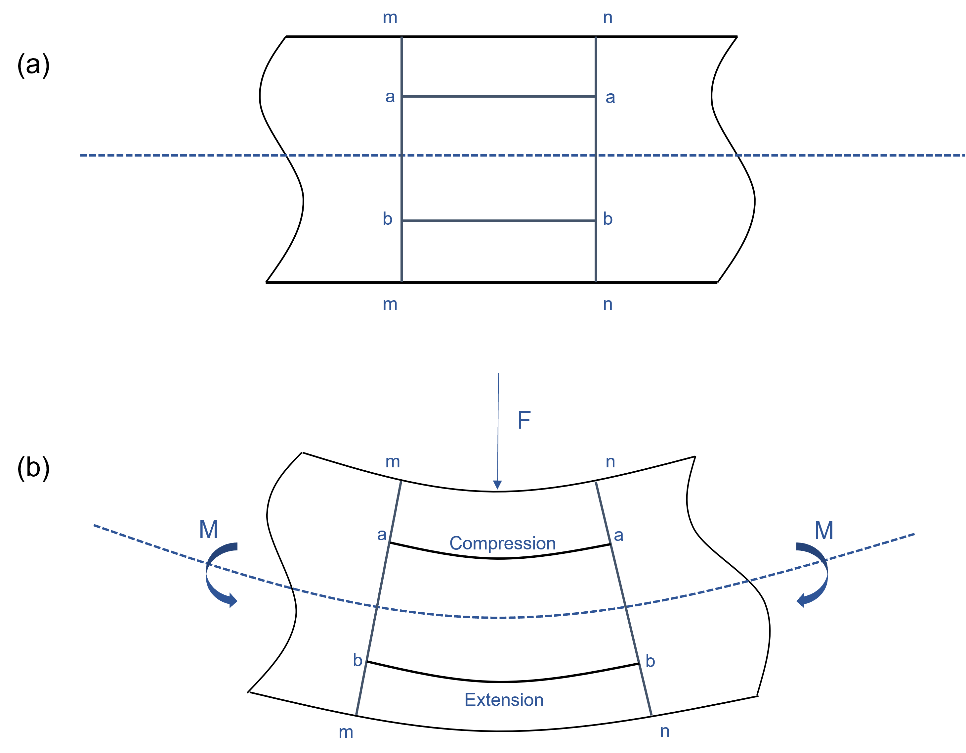


**Figure S1.9**: (a) The beam in its initial undeformed configuration (b)Subsequent deformation of the beam. The dotted line delineates the neutral layer.

In constructing a mathematical model to describe the bending behavior of this structure, our main objective revolves around understanding the relationship between the applied load and the resulting deformation, with a specific focus on the notch width. A significant advantage in analyzing beams is the ability to establish a neutral layer within each beam with uniform material distribution. During the elastic bending of the beam, this neutral layer maintains constant dimensions. Meanwhile, the top and bottom surface layers undergo extension and compression, respectively, as illustrated in **Figure S1.9**.

Leveraging the inherent properties of the neutral layer, we can readily derive numerous valuable geometric relationships. When considering an arbitrary section of the beam, we define a segment  $\bar{bb}$ situated at a distance y below the neutral layer  $\bar{OO}$. Both the neutral layer and segment have an original length of $dx$ before bending. We can express this relationship as follows,

|  | $\overline{bb}=dx=\overline{OO}$ | (S4) |
| --- | --- | --- |

When the beam deforms under bending, the length of the neutral layer  $\bar{OO}$ remains unchanged based on its properties,

|  | $dx=\bar{OO}=\breve{OO}=\rho d\theta$ | (S5) |
| --- | --- | --- |

Where $\rho$ is the bent beam’s radius of curvature with angle $d\theta$. Then, for the case of the interested segment $\bar{bb}$, we have

|  | $\breve{bb}=(\rho+y)d\theta$ | (S6) |
| --- | --- | --- |

Here, $\breve{bb}$ and $\breve{OO}$ represent the length of the segment  $\bar{bb}$ and  $\bar{OO}$ respectively. When the material is bent under compression. Consequently, the strain ($\epsilon$) can be defined as follows,

|  | $\epsilon=\frac{\left( \rho+y \right)d\theta-\rho d\theta}{\rho d\theta}=\frac{y}{\rho}$ | (S7) |
| --- | --- | --- |

Expanding on the geometric relationships we have established, let's further explore the fundamental physical connections between stress $\sigma$ and strain $\epsilon$. These relationships are crucial foundations for comprehending the structural behavior of materials when subjected to bending. Understanding how stress and strain interrelate is fundamental in analyzing and predicting the behavior of materials and structures under various mechanical conditions, particularly bending.


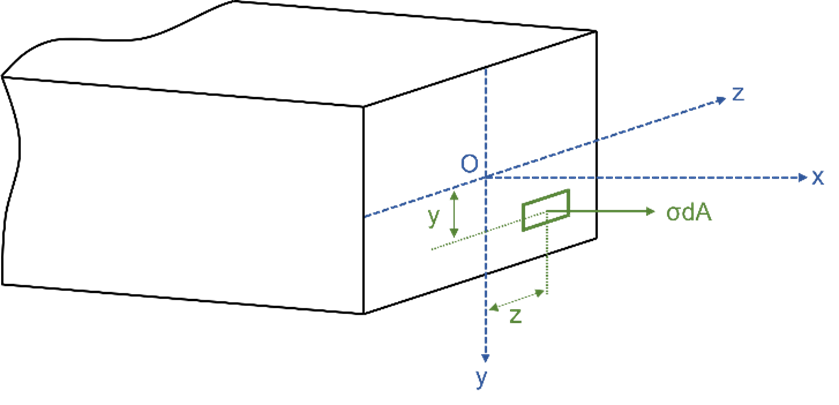


**Figure S1.10**: Examining the cross-section of the beam, we set the stage for formulating physical relationships by establishing a Cartesian coordinate system centered within the beam's cross-section. Within this framework, we consider an infinitesimal area $dA$.

We start by defining a Cartesian coordinate system, as shown in **Figure S1.10**, using the neutral layer as the reference, within the cross-section of the beam. The origin is placed at the center of the cross-section. Considering an infinitesimal area $dA$, we determine its offsets to the $y$ and $z$ axes as $y$ and $z$ respectively. With this foundation, we proceed to derive fundamental relationships that illuminate the connections between stress, strain, and the geometry of the beam. These relationships are essential in understanding how stress and strain manifest in the structure in relation to its geometry.

|  | $\sigma=E\epsilon$ | (S8) |
| --- | --- | --- |

|  | $\Rightarrow\sigma=E\frac{y}{\rho}\propto y$ | (S9) |
| --- | --- | --- |

|  | $\sigma=\frac{My}{I_{z}}$ | (S10) |
| --- | --- | --- |

In the above expressions, $E$ represents the Young’s Modulus of the beam, $\rho$ stands for the radius of curvature of the deformation, and $I_{z}$ defines the 2nd Moment of Inertia (area moment of inertia) at the beam’s cross-section.

To prepare for modeling the deformation of a notched beam, it's crucial to initially understand the deformation characteristics of an unnotched beam. Let's define an arbitrary beam with a length $L$, width $w$, and height $h$, as shown in **Figure S1.11**.

This unnotched beam acts as a fundamental reference, providing insights into the behavior of beams without notches. Later, we can contrast this with notched beams to comprehend the influence of notches on deformation properties.


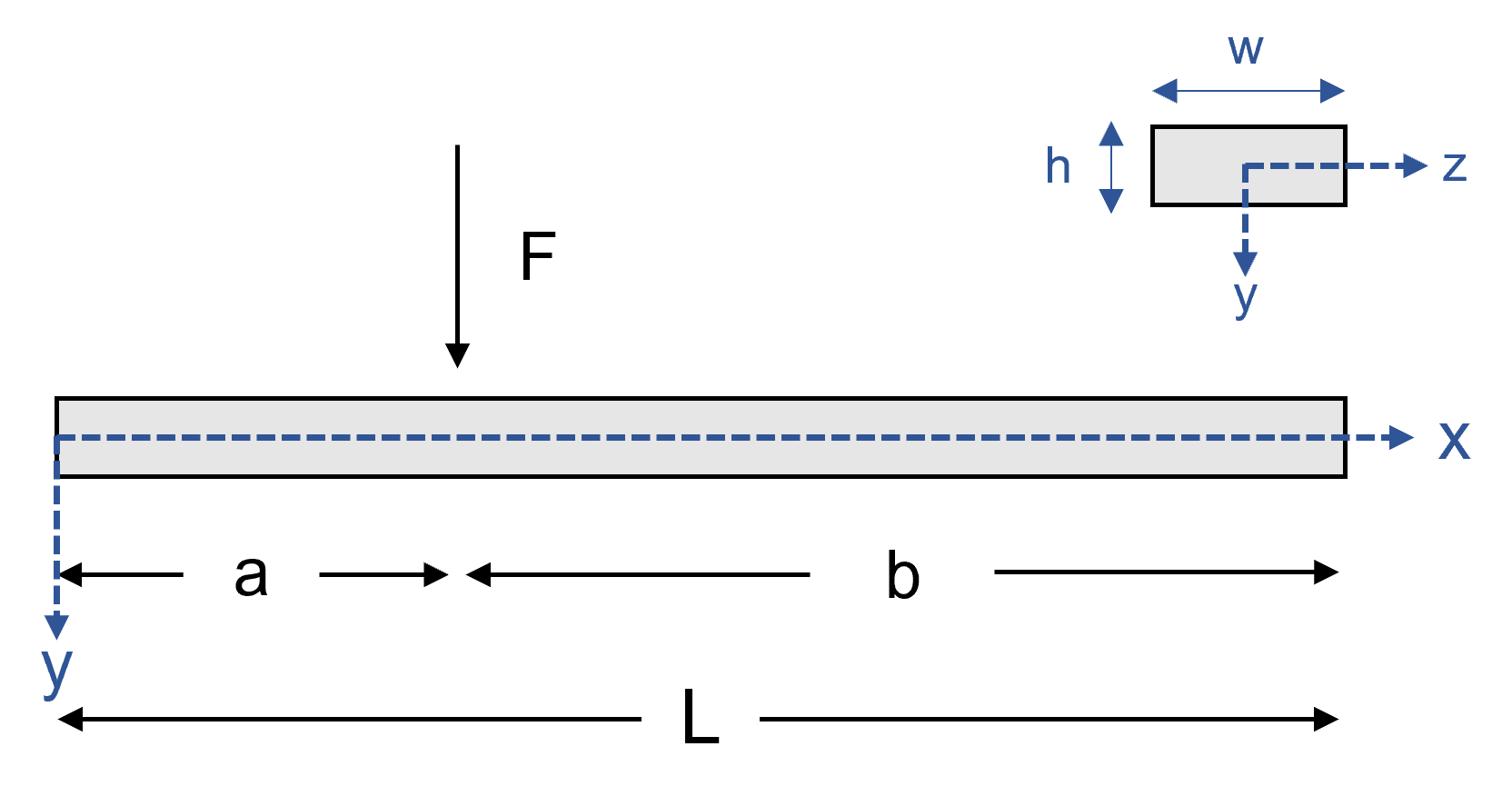


**Figure S1.11**: 3D beam of length L, width w, and height h under applied load F. A Cartesian coordinate system is attached as shown.

When a load $F$ is applied, the beam undergoes deformation. If we select an arbitrary segment $d_{0}$ on the opposite side of where the load is applied, it will extend by $\Delta d_{0}$, as illustrated in **Figure S1.12**.


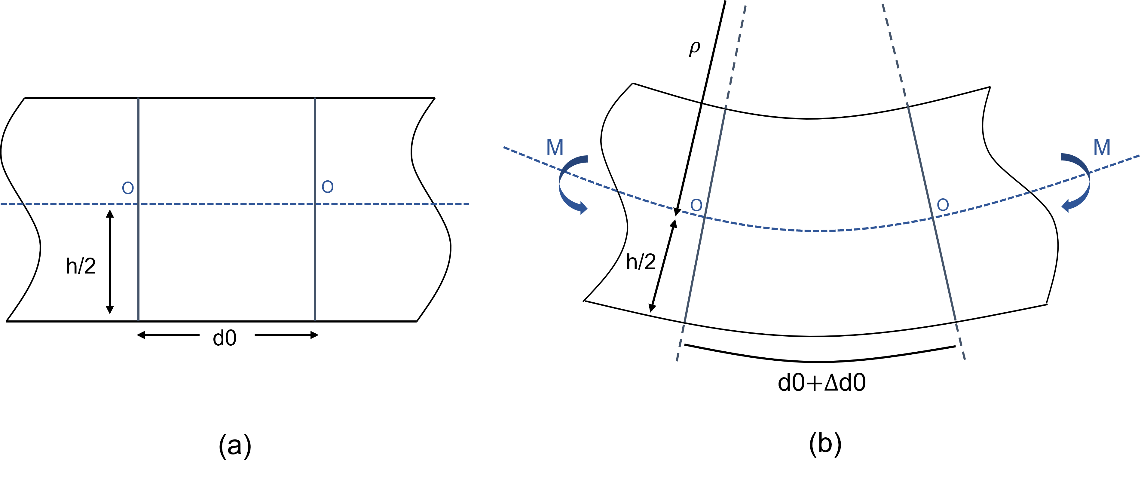


**Figure S1.12**: (a) Unnotched beam in its undeformed shape with projected notch width $d_{0}$. (b) Unnotched beam undergoing bending with projected deformed notch width $d_{0}+\Delta d_{0}.$

Referring back to the geometric and physical relationships introduced in the previous section, we can establish the following relationships:

|  | $\breve{d_{0}+\Delta d_{0}}=\left( \rho+\frac{h}{2} \right)d\theta$ | (S11) |
| --- | --- | --- |

|  | $d_{0}=\overline{OO}=\breve{OO}=\rho d\theta$ | (S12) |
| --- | --- | --- |

From these, we can calculate the strain in this case be,

|  | $\epsilon_{0}=\frac{{\left( d_{0}+\Delta d_{0} \right)-d}_{0}}{d_{0}}=\frac{\Delta d_{0}}{d_{0}}=\frac{h}{2\rho}$ | (S13) |
| --- | --- | --- |

Finally, we can proceed to calculate the deformation (in this case, extension) of $d_{0}$ using the following expressions:

|  | $\frac{6M}{wEh^{2}}=\frac{\Delta d_{0}}{d_{0}}$ | (S14) |
| --- | --- | --- |

|  | $\Rightarrow\Delta d_{0}=\frac{6Md_{0}}{wEh^{2}}$ | (S15) |
| --- | --- | --- |

**1.4 DEFORMATION MODELING OF LOADING NOTCHED BEAM**

Saint-Venant’s principle provides valuable insights by indicating that the stress state remains nearly unchanged at a distance approximately equal to the linear dimension of the loaded area. In cases where a disturbance, such as holes or notches, is located far from the loaded region, stress computation assumes an even load distribution. The critical distance is associated with the size of the entire boundary if the load is confined. In the context of finite element method (FEM) analysis, when the disturbance is near the load, its influence is limited by the requirement for a well-defined load distribution based on appropriate assumptions of redistribution.

In our specific scenario, we can apply Saint-Venant’s principle to infer that stresses on a boundary considerably distant from the applied load remain relatively unaffected. The alterations in stress and strain primarily occur in the vicinity of the load application regions. Given that the notches are positioned on the opposite side from where the load is applied and considering the thinness of the notches relative to the beam's thickness, we can confidently employ Saint-Venant’s Principle in our analysis.

To simplify our analysis leveraging Saint-Venant’s principle, we opt to consider the regions immediately surrounding the notch as stress-free areas. We can start from the central location (Location C), which proves to be the most convenient for analysis due to its symmetrical properties.

Here, we represent the stress-free area as an isosceles triangle with a height of $\alpha$ and a base length of $2\alpha$, as shown in **Figure S1.13**. This approach simplifies computations and aligns with the Saint-Venant’s principle's application.


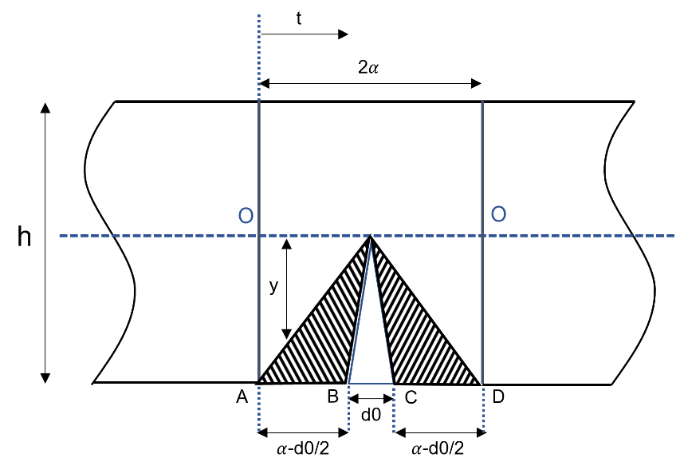


**Figure S1.13**: Simplifying the analysis of the notched beam utilizing Saint-Venant’s Principle at the central location.

In this context, $d_{0}$ represents the original notch width situated at the center of the expanded configuration. According to Saint-Venant’s principle, the expanded sections (shaded areas) will experience negligible deformation during the beam's bending. Consequently, the extension in the expanded configuration (segment $\bar{AD}$) is equal to the extension in the original configuration (segment $\bar{BC}$), denoted as $\Delta d$.

Extending from the geometric and physical relationships elucidated in previous sections, we now proceed to derive the following expressions to delineate this relationship:

|  | $y=\alpha-t$ | (S16) |
| --- | --- | --- |

|  | $\Rightarrow\epsilon_{1}E=\frac{M_{C}y}{I_{z}}=\frac{M_{C}\left( \alpha-t \right)}{\frac{1}{12}w{E\left( 2\alpha-t \right)}^{3}}$ | (S17) |
| --- | --- | --- |

|  | $\Rightarrow\epsilon_{1}=\frac{12M_{C}(\alpha-t)}{w{E\left( 2\alpha-t \right)}^{3}}$ | (S18) |
| --- | --- | --- |

Utilizing the fundamental definition of strains as the ratio of deformation to the original length, we apply basic principles of calculus to express it in terms of an arbitrary length $t$.

|  | $\epsilon=\lim_{t\to0} (\Delta t)$ | (S19) |
| --- | --- | --- |

Through integration across the entire expanded area depicted in **Figure S1.14**, resembling an isosceles triangle, we can determine the deformation of the notch width. This integration process is crucial for quantifying the extent of deformation experienced by the notch width as the beam undergoes bending.


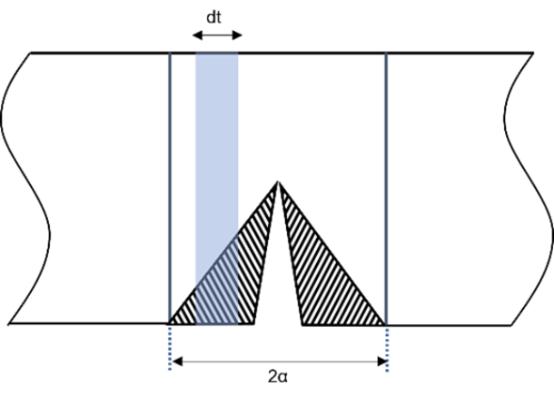


**Figure S1.14**: When integrating over the entire expanded area, represented by an isosceles triangle, we can obtain the deformation of the notch width. This integration process allows us to quantify the extent of deformation experienced by the notch width during the beam's bending.

Furthermore, to ensure smooth integration and avoid discontinuity at the tip of the triangle, we consider integration over a symmetrical area. This allows us to perform the following operations to derive the deformation:

|  | $\Delta d_{C}=\int_{0}^{2\alpha} \epsilon_{1}dt=2\int_{0}^{\alpha} \epsilon_{1}dt$ | (S20) |
| --- | --- | --- |

|  | $\Rightarrow\Delta d_{C}=\frac{3M_{C}}{wE\alpha}$ | (S21) |
| --- | --- | --- |

When analyzing intermediate locations (A, B, D, and E) that are off-center, a notable asymmetry becomes evident regarding the stress-free areas. These stress-free areas closely resemble the shape of the respective moment diagrams at each location.

Saint-Venant's principle indicates that the stress-free area is inversely related to deformation, where smaller stress-free areas experience more significant deformation and vice versa. Therefore, during integration, similar to the central location, we should integrate using a horizontal mirror image of the corresponding moment diagram to accurately capture the varying deformation across these off-central locations. This observation emphasizes the importance of considering the shape of stress-free areas in accurately analyzing and interpreting the beam's behavior at different positions.

We initiate our analysis with intermediate location A (with similar considerations applicable to location E due to symmetry), focusing on the geometric representation of the stress-free area. Here, we model the stress-free area's geometry as a horizontal mirror image of the moment diagram. This stress-free area takes the form of a scalene triangle characterized by a height of $\alpha$ and a base length of $2\alpha$, as depicted in **Figure S1.15** through the shaded regions.


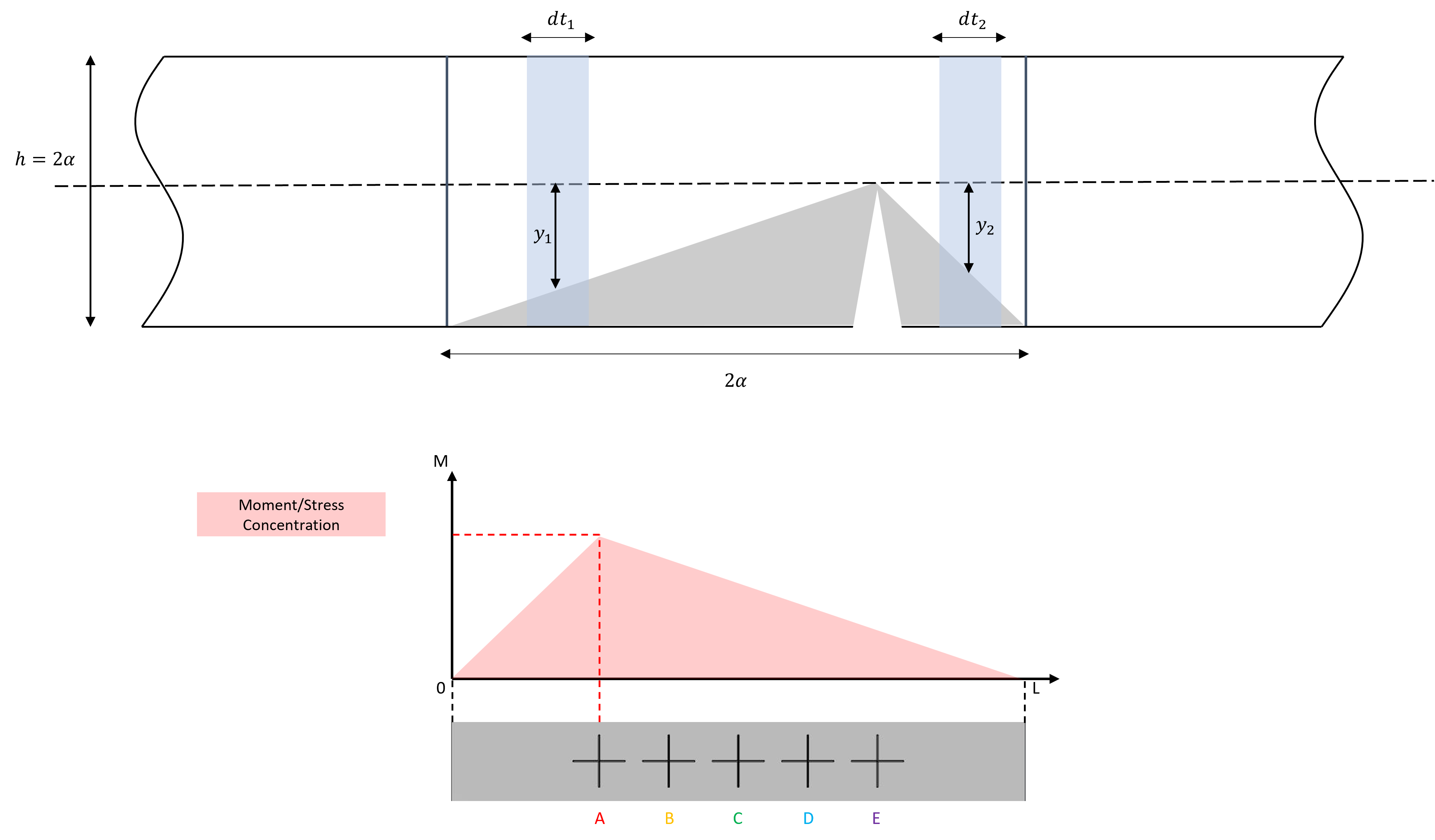


**Figure S1.15**: Representation of the stress-free area at intermediate location A. Unlike the central location, the stress-free area is not an isosceles triangle. Instead, it is shaped in accordance with the corresponding moment diagram. The stress-free area's geometry reflects the moment diagram, showcasing a shape that accurately captures the stress-free region for this location.

Expanding on the previously established geometric and physical relationships, we now derive expressions that encapsulate this relationship, emphasizing the introduction of two parameters, $t_{1}$ and $t_{2}$. This represents a departure from the formulation for the central location C, which involved only a single parameter. Additionally, we incorporate the specific geometry of the notch into our analysis. Location A is positioned at 4.25mm from the closer boundary, relative to the entire beam, which has a length of 16.5mm. Consequently, this gives the moment $M_{A}=\frac{833}{4356}FL$.

|  | $y_{1}=\alpha-\frac{165}{245}t_{1} y_{2}=\frac{165}{85}t_{2}$ | (S22) |
| --- | --- | --- |

|  | ${\epsilon_{A}}_{1}=\frac{M_{A}y_{1}}{I_{z}E}=\frac{12M_{A}(\alpha-\frac{165t_{1}}{245})}{wE\left( 2\alpha-\frac{165t_{1}}{245} \right)^{3}}$ | (S23) |
| --- | --- | --- |

|  | ${\epsilon_{A}}_{2}=\frac{M_{A}y_{2}}{I_{z}E}=\frac{12M_{A}(\frac{165t_{1}}{85})}{wE\left( \alpha+\frac{165t_{1}}{85} \right)^{3}}$ | (S24) |
| --- | --- | --- |

By adhering to strain's fundamental definition as deformation relative to the original length and utilizing principles of calculus, we perform integration across the shaded area in **Figure S1.15** to calculate the deformation of the notch width at this intermediate location. To handle the discontinuity at the triangle's peak, we conduct two separate integrations. These separate integrations are vital for accurately calculating deformations at the left and right edges of the notch.

|  | $\Delta d_{A}=\int_{0}^{\frac{245\alpha}{165}} {\epsilon_{A}}_{1}dt+\int_{0}^{\frac{85\alpha}{165}} {\epsilon_{A}}_{2}dt$ | (S25) |
| --- | --- | --- |

|  | $\Delta d_{A}=\Delta d_{A_{1}}+\Delta d_{A_{2}}=\frac{49M_{A}}{22wE\alpha}+\frac{17M_{A}}{22wE\alpha}=\frac{3M_{C}}{wE\alpha}$ | (S26) |
| --- | --- | --- |

Lastly, shifting our focus to intermediate location B (with analogous considerations applicable to location D due to symmetry), we employ a method similar to our approach for formulating deformation at intermediate locations A and E. Here, we model the stress-free area's geometry as a horizontal mirror image of the moment diagram. This stress-free area takes on the configuration of a scalene triangle, characterized by a height of $\alpha$ and a base length of $2\alpha$, as depicted in **Figure S1.16** through the shaded regions.


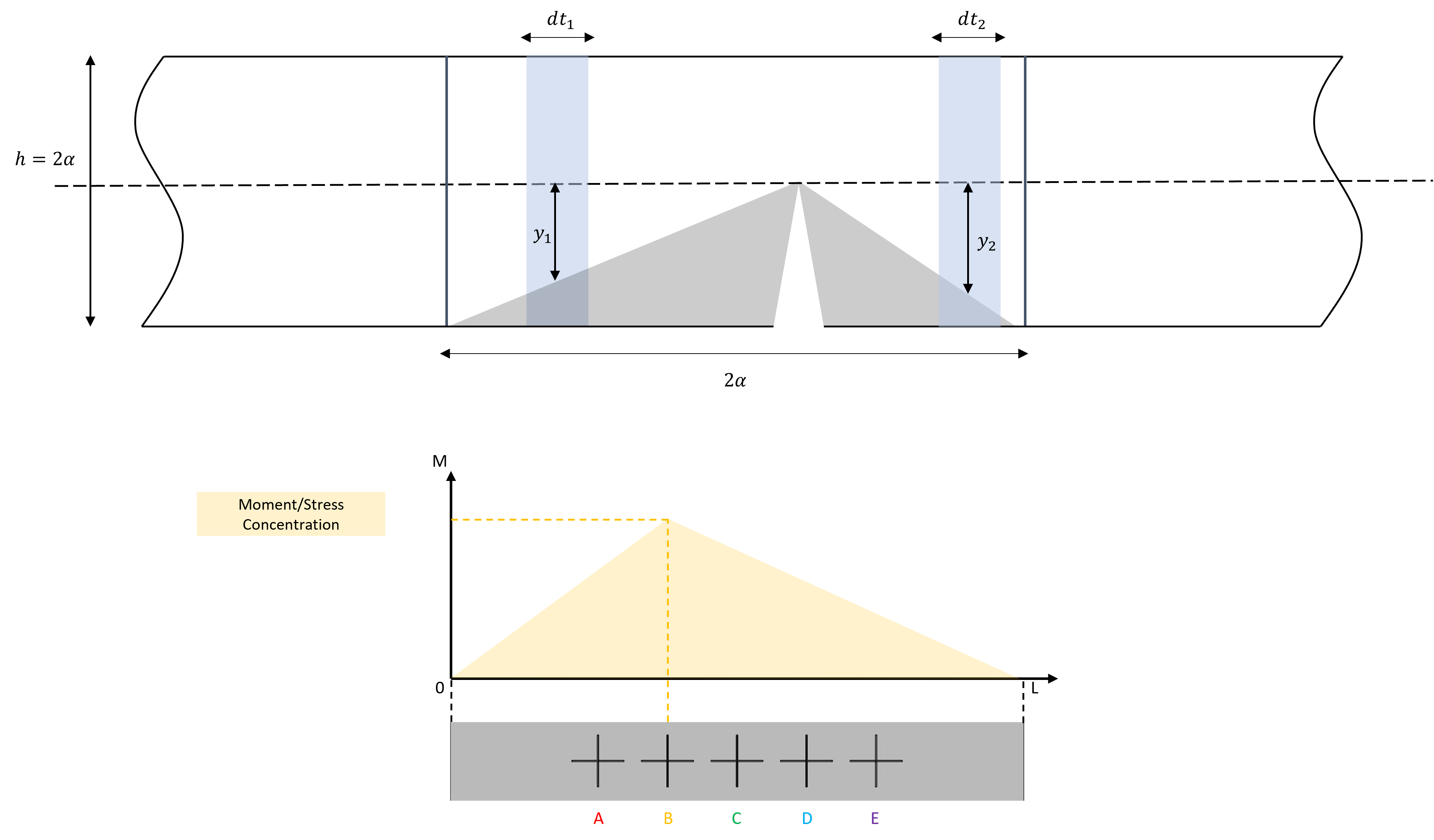


**Figure S1.16**: Graphical representation of the stress-free area at intermediate location B. This stress-free area conforms to the shape dictated by the corresponding moment diagram, mirroring its configuration. The illustration vividly portrays the accurate delineation of the stress-free region specific to this location, effectively capturing the structural behavior in this region.

Building upon established geometric and physical relationships, we proceed to derive expressions that encapsulate this relationship. Once again, we introduce two parameters, $t_{1}$ and $t_{2}$, while assimilating the specific geometry of the notch into our analytical framework. Notably, location B is positioned at 5.75mm from the nearer boundary, within the context of the beam that spans a length of 16.5mm. Consequently, this determination yields $M_{B}=\frac{989}{4356}FL$.

|  | $y_{1}=\alpha-\frac{165}{215}t_{1} y_{2}=\frac{165}{115}t_{2}$ | (S27) |
| --- | --- | --- |

|  | ${\epsilon_{B}}_{1}=\frac{M_{B}y_{1}}{I_{z}E}=\frac{12M_{B}(\alpha-\frac{165t_{1}}{215})}{wE\left( 2\alpha-\frac{165t_{1}}{215} \right)^{3}}$ | (S28) |
| --- | --- | --- |

|  | ${\epsilon_{B}}_{2}=\frac{M_{B}y_{2}}{I_{z}E}=\frac{12M_{B}(\frac{165t_{1}}{115})}{wE\left( \alpha+\frac{165t_{1}}{115} \right)^{3}}$ | (S29) |
| --- | --- | --- |

Following the same procedure, we perform integration over the shaded region in **Figure S1.16**. To address the discontinuity at the triangle's peak, we conduct two separate integrations for deformation calculations at the left and right edges. This approach ensures accurate deformation calculations for both sides of the notch.

|  | $\Delta d_{B}=\int_{0}^{\frac{215\alpha}{165}} {\epsilon_{B}}_{1}dt+\int_{0}^{\frac{115\alpha}{165}} {\epsilon_{B}}_{2}dt$ | (S30) |
| --- | --- | --- |

|  | $\Delta d_{B}=\Delta d_{B_{1}}+\Delta d_{B_{2}}=\frac{43M_{B}}{22wE\alpha}+\frac{23M_{B}}{22wE\alpha}=\frac{3M_{C}}{wE\alpha}$ | (S31) |
| --- | --- | --- |

References

[1] R. v. Mises, “On Saint Venant’s principle,” *Bulletin of the American Mathematical Society*, vol. 51, no. 8, pp. 555–562, 1945, doi: 10.1090/S0002-9904-1945-08394-3.

Supplementary Figure S2


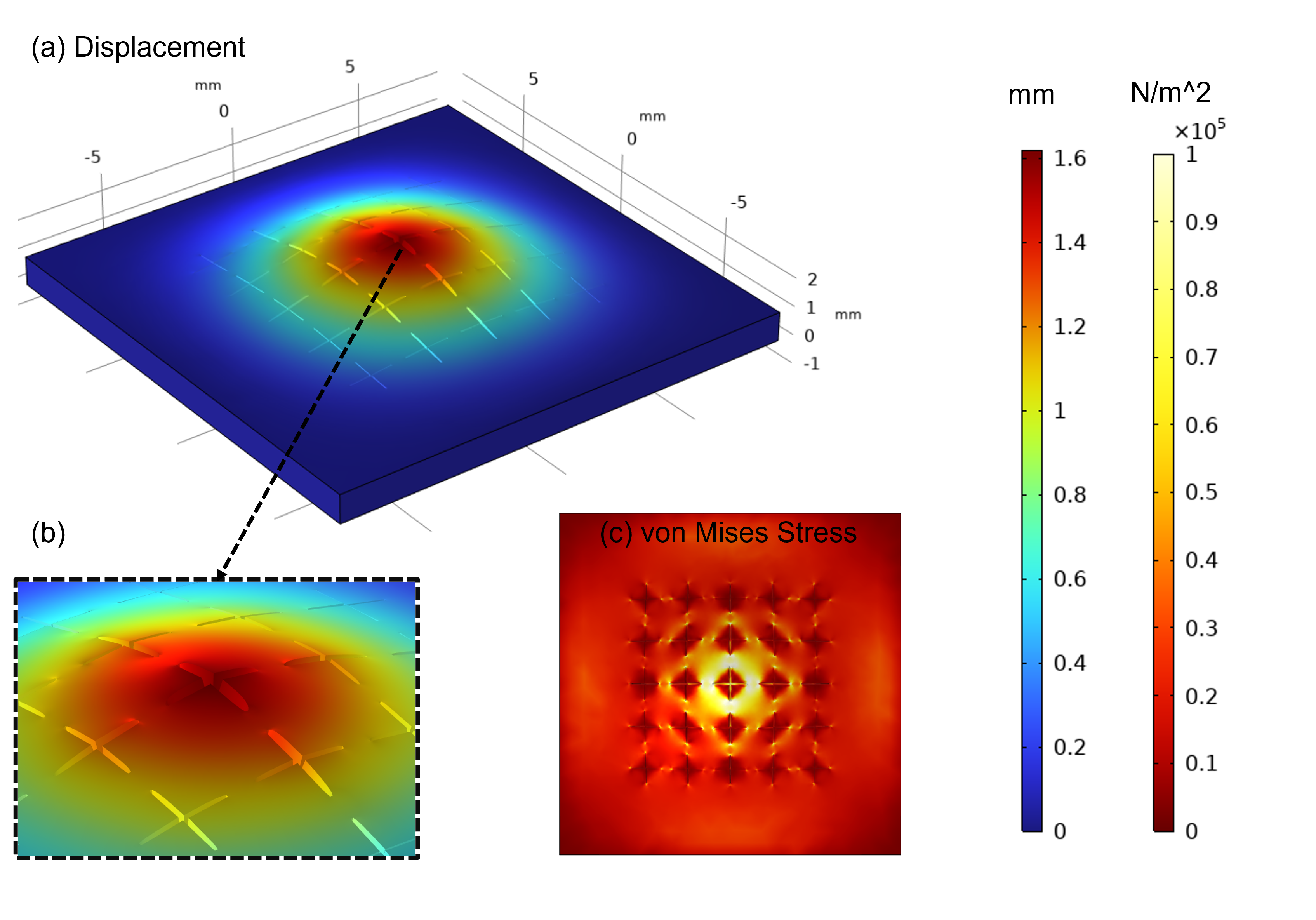


**Figure S2**: Finite element analysis of (a) deformation and (b) stress distribution with a force of 60 mN applied at the bottom centre (3, 3) of the sensor along the y-direction^[[1]](#footnote-2)^ (diameter of contact area: 1 mm).

Supplementary Figure S3


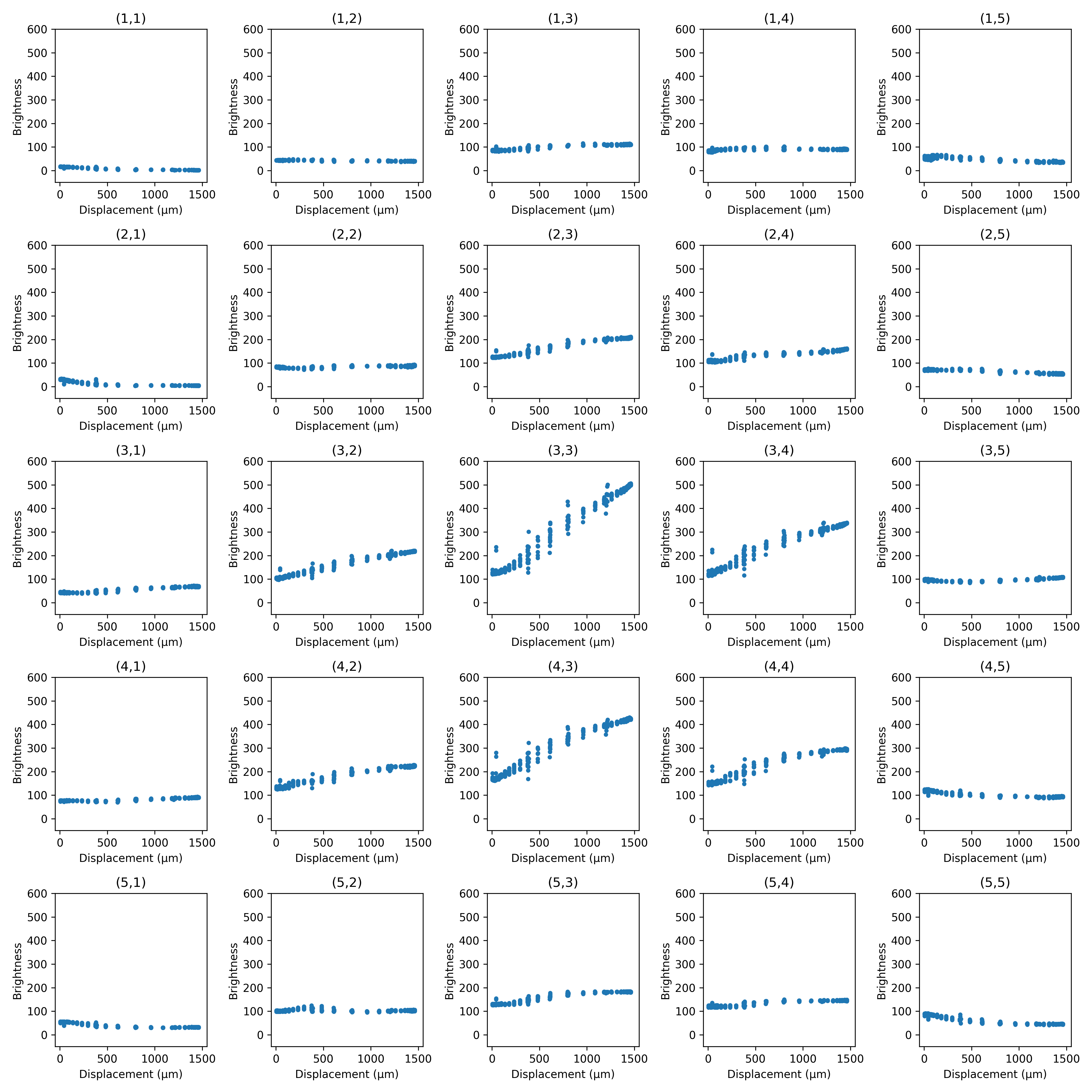


**Figure S3**: Variation of brightness with different displacements (0-1.5 mm) at 25 regions. The contact point was located at the center region (3,3). Analysis was performed on 300 continuous frames recorded at 30 FPS. Each subplot shows the brightness response of a specific region as a function of displacement.

Supplementary Figure S4


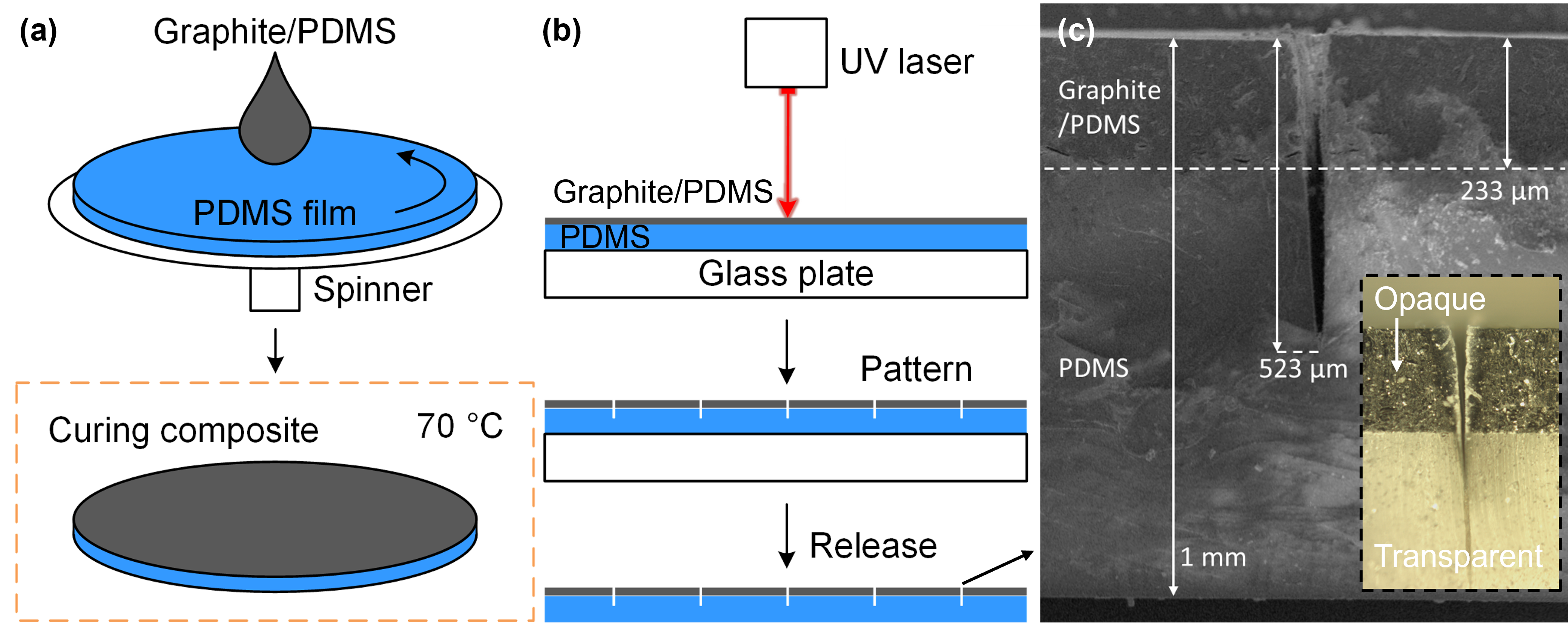


**Figure S4**: Fabrication processes and characterisation. (a) preparation of the film with the spinning coating process. (b) micromachining with high-precision laser cutting.^[[2]](#footnote-3)^ (c) Characterisation of a micro trench with SEM and optical microscope.

Supplementary Figure S5


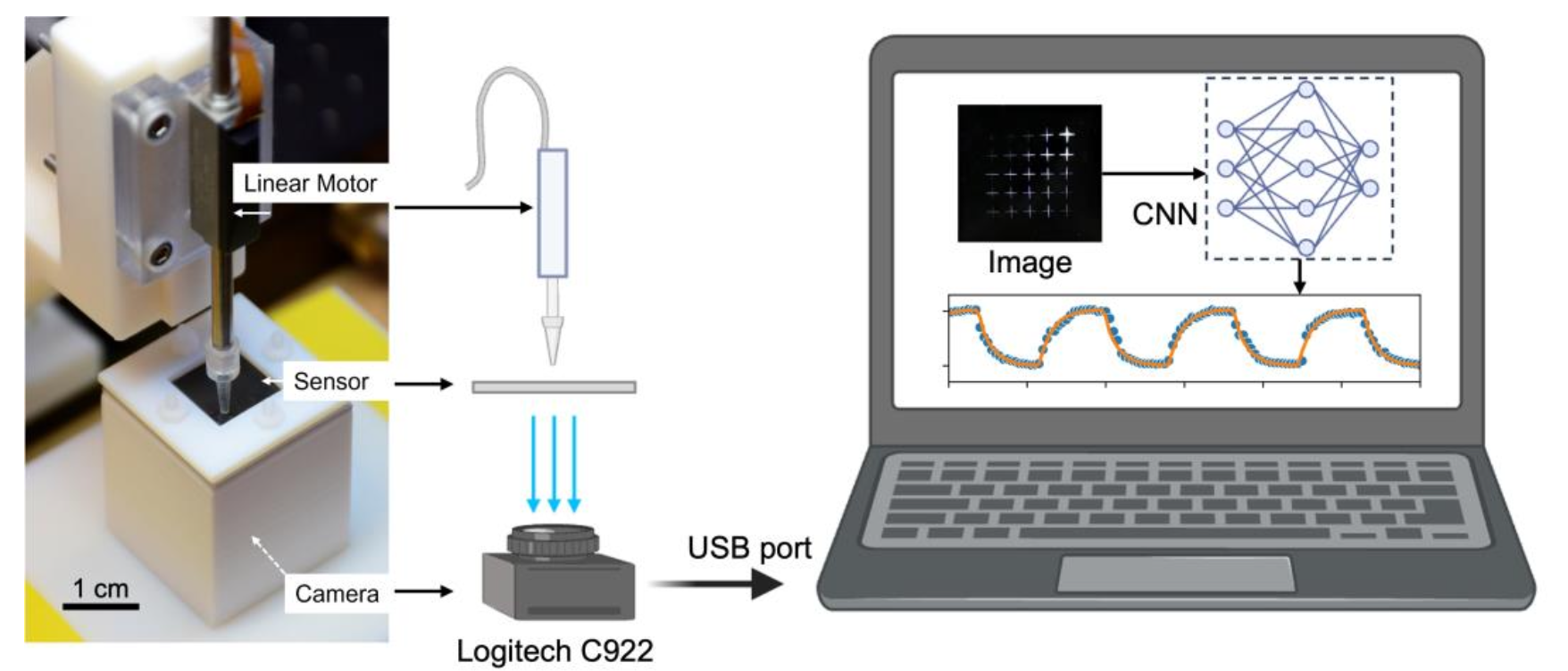


**Figure S5**: Experimental set-up. The setup includes a linear motor (Faulhaber LM083004001), a sensor (Micro-VBTS), and a camera (Logitech C922 camera).^[[3]](#footnote-4)^ Diameter of contact area: 1 mm.

Supplementary Figure S6


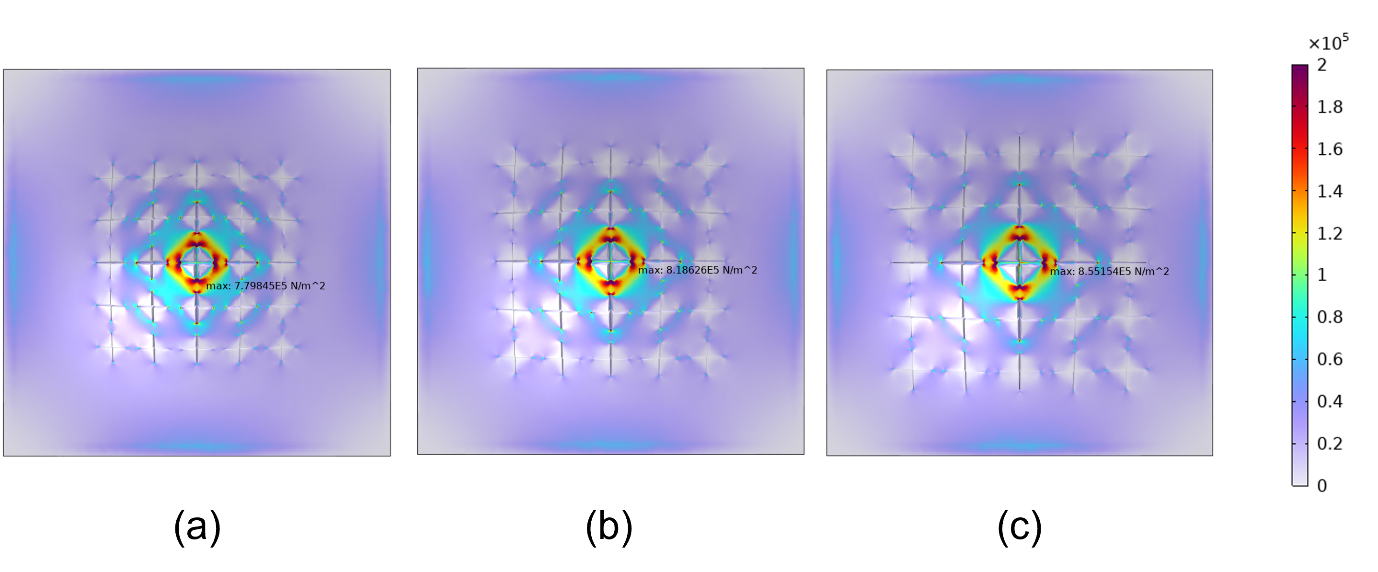


**Figure S6**: von Mises stress distribution for cross-shaped notches with different arm lengths (a) 1.25 mm arm length: max stress = 7.80 x 10⁵ N/m² (b) 1.50 mm arm length (our design): max stress = 8.19 x 10⁵ N/m² (c) 1.75 mm arm length: max stress = 8.55 x 10⁵ N/m²

Note: To investigate the influence of the cross-trench geometry, we simulated two additional models with arm lengths of 1.25 mm and 1.75 mm, keeping all other parameters consistent with our original design (1.50 mm arm length). The results, shown in the Figure 1, indicate a clear trend: the maximum von Mises stress at the centre of the applied force increases with the length of the micro-trench arms. While a longer arm (1.75 mm) produces higher stress and could theoretically lead to higher sensitivity, it also brings the material closer to its plastic deformation or failure limits, potentially compromising long-term durability. Conversely, a shorter arm (1.25 mm) results in a lower stress response, which might reduce the sensor's sensitivity to small forces.

Supplementary Figure S7


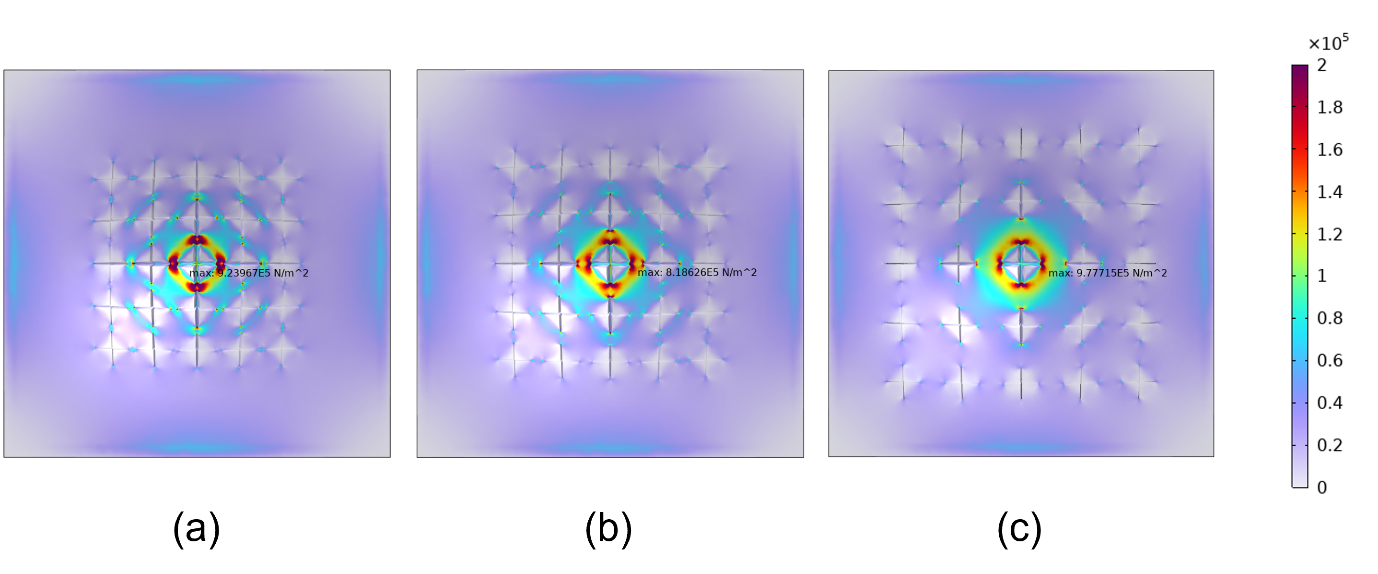


**Figure S7**: von Mises von Mises stress distribution for cross-shaped notches with different spacing (a) 0.25 mm gap: max stress = 8.30 x 10⁵ N/m² (b) 0.50 mm gap (our design): max stress = 8.19 x 10⁵ N/m² (c) 1.00 mm gap: max stress = 9.78 x 10⁵ N/m²

Note: We analyzed the effect of the spacing between adjacent cross-trenches. We simulated configurations with gaps of 0.25 mm and 1.00 mm and compared them to our original design, which has a 0.50 mm gap. Interestingly, the relationship between spacing and maximum stress is not monotonic. Both the smaller and larger gaps produced higher maximum stress compared to our chosen design. This non-monotonic behaviour highlights the complex interplay between adjacent microstructures. At 0.25 mm (smaller gap): The structures are highly coupled. The material between the trenches is more constrained, and the deformation fields of neighbouring crosses interact strongly, leading to an increase in localized stress. At 1.00 mm (larger gap): The central cross-trench becomes more structurally isolated. It receives less mechanical support from its neighbours, forcing it to bear a larger portion of the applied load, which results in higher stress.

Supplementary Figure S8

**
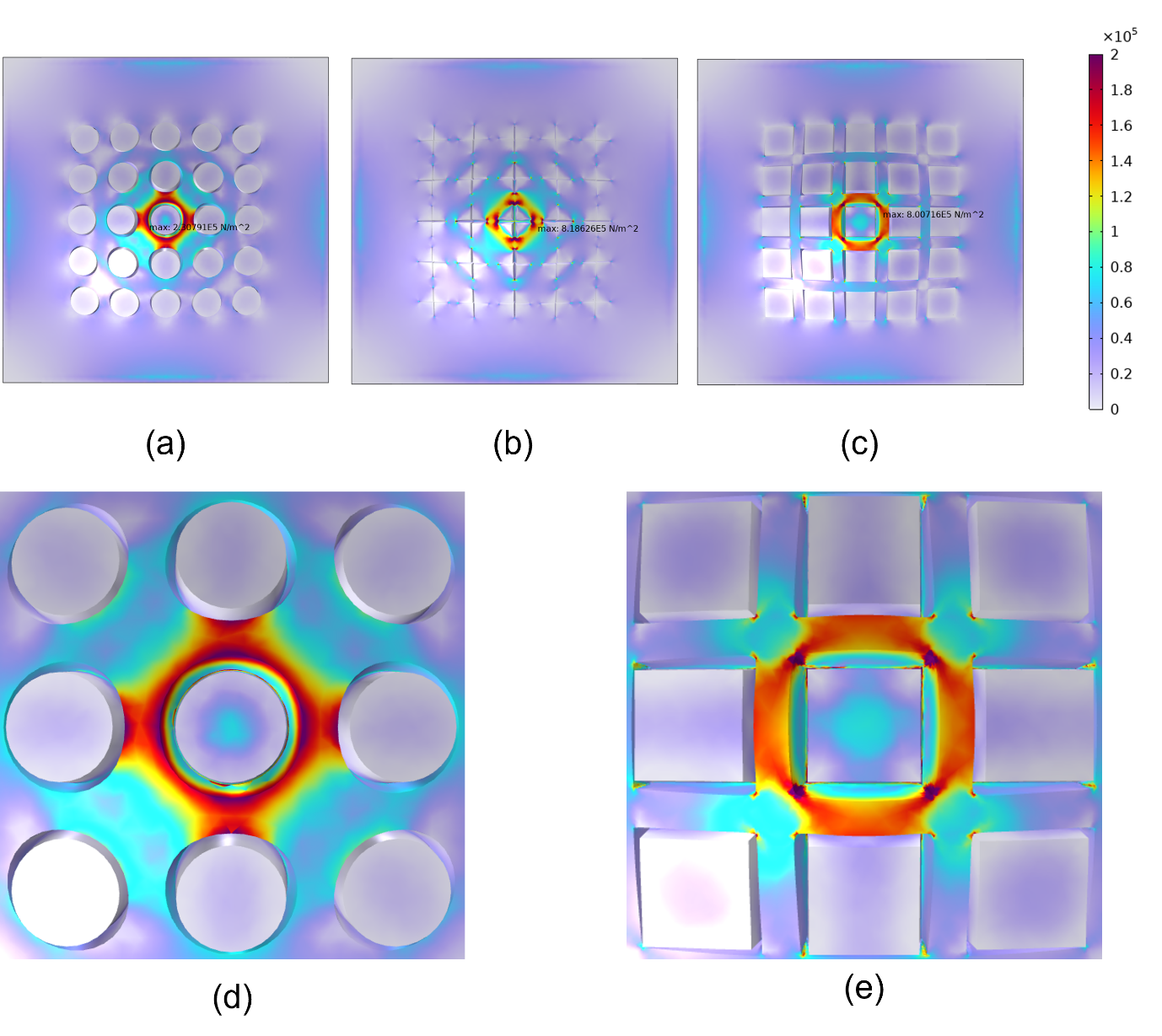
**

**Figure S8**: FEA simulation of alternative microstructure shapes (von Mises stress distribution, unit: N/m2). (a) and (d): circular trench. (b) Cross-trench (our design). (c,e) square trench.

**Supplementary Figure S9**


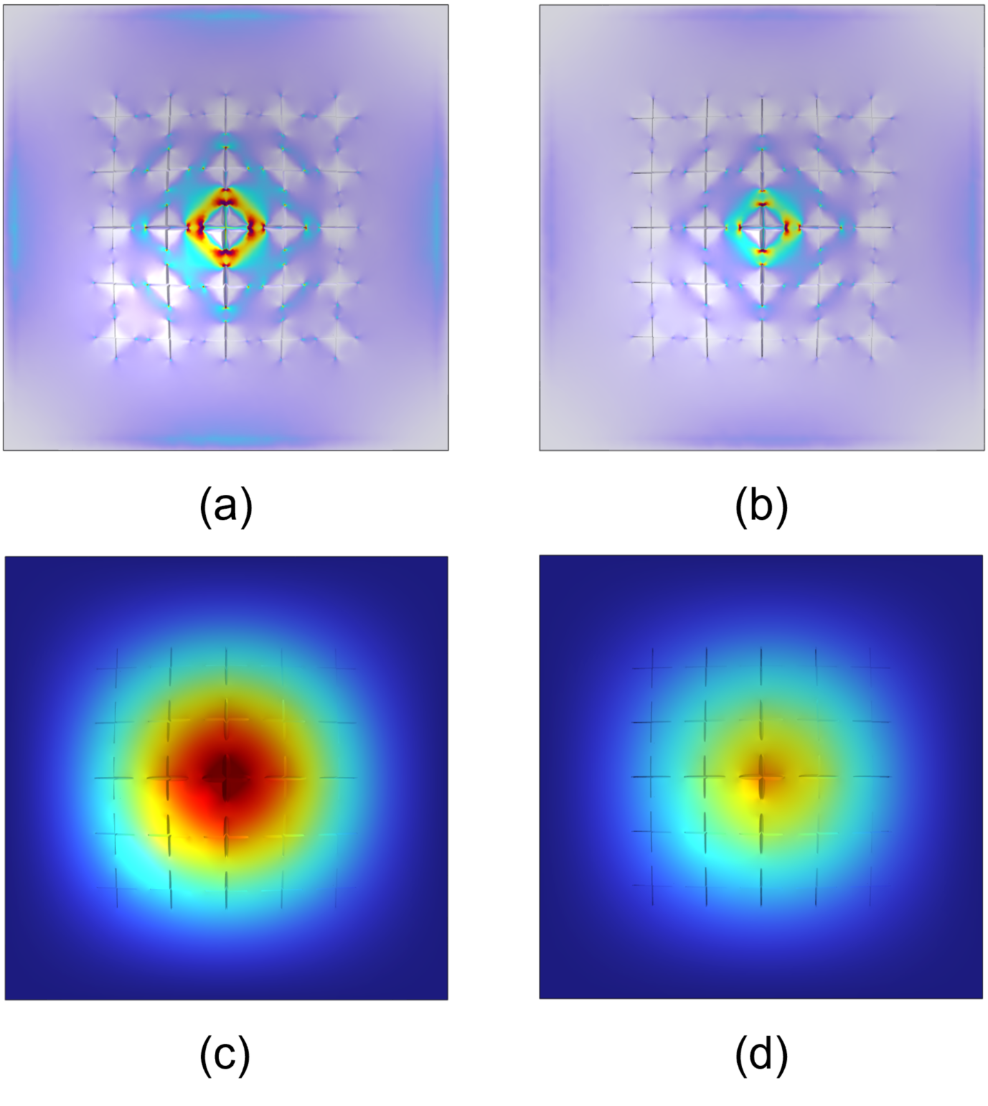


**Figure S9**: Comparison of von Mises stress distribution and displacements under an 80 mN normal force and an 80 mN force applied at a 45-degree angle to the normal direction, tilted toward the right. (a) von Mises stress distribution under an 80mN normal force (b) von Mises stress distribution under an 80mN 45-degree off-axis force (c) Displacement an 80mN normal force (d) Displacement under an 80mN 45-degree off-axis force.

Note: The simulation reveals that the introduction of a shear force fundamentally changes the sensor’s response from a symmetrical to a distinctly asymmetrical pattern. While the normal force produces concentric stress and displacement fields (Fig. S9a, c), the combined normal and shear load results in a stress distribution (Fig. S9b) and displacement field (Fig. S9d) that are clearly skewed in the direction of the shear force.

**Supplementary Figure S10**


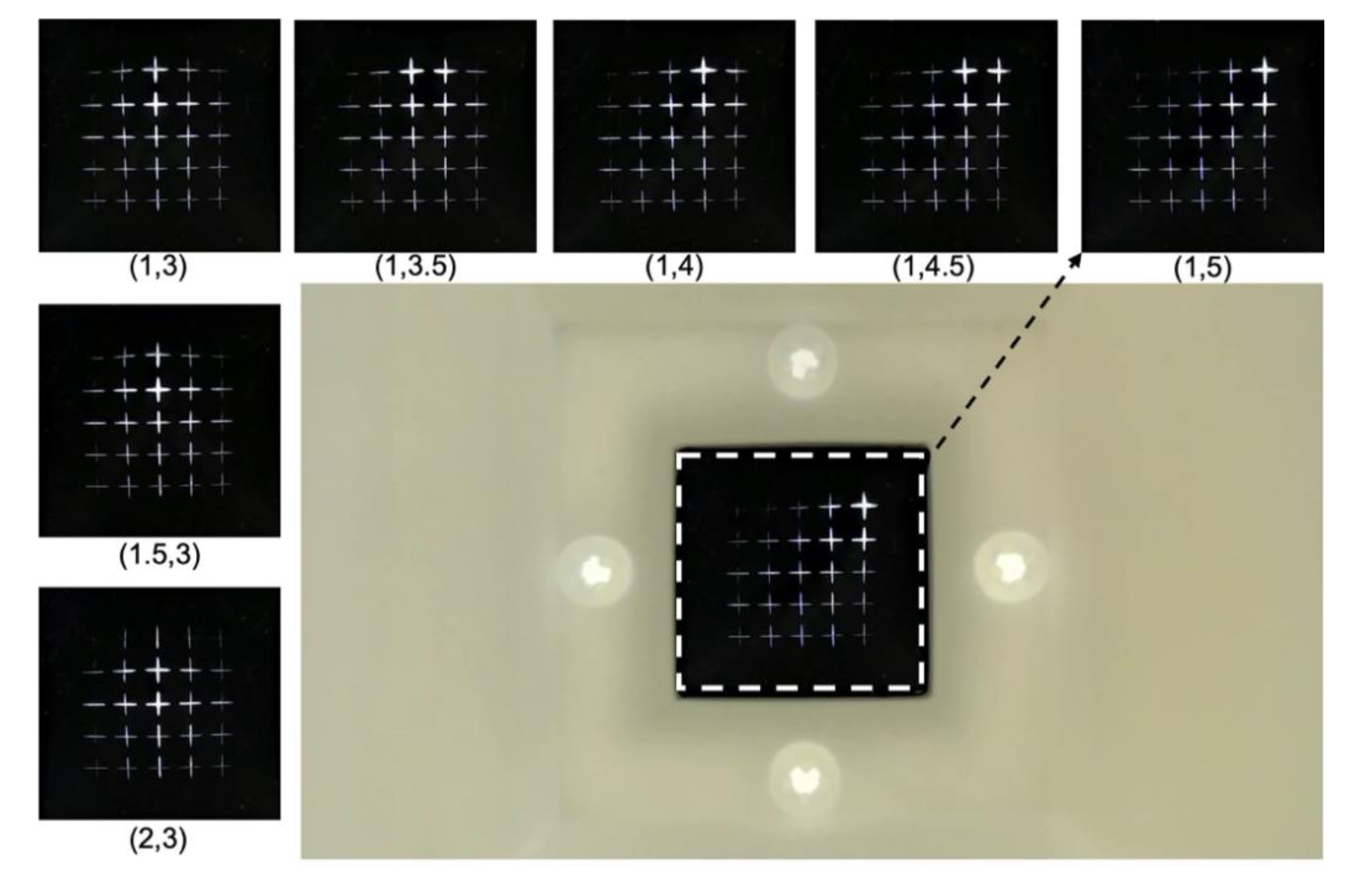


**Figure S10**: Sub-grid localization capability of the Micro-VBTS sensor. Seven test points were selected along a path from (1,5) to (1,3) to (2,3) with 0.5-grid spacing intervals. Among them, three points lie between the fabricated cross-shaped structures. The corresponding sensor images show distinguishable patterns, indicating the system’s potential to resolve positions beyond the discrete calibration grid. These results demonstrate that the sensor's localisation accuracy is not limited by the density of grid. The 5×5 calibration grid defines evaluation coverage rather than intrinsic spatial resolution.

Supplementary

Table S1

**Table S1**: Metrics for evaluating model performance

| Metrics  Model | z-coordinate/displacement (mm) | | | |
| --- | --- | --- | --- | --- |
|  | MSE | RMSE | MAE | R² |
| CNN_1 | 0.002225 | 0.047166 | 0.030523 | 0.988868 |
| CNN_3 | 0.002045 | 0.045216 | 0.027137 | 0.989769 |
| CNN_5 | 0.002002 | 0.044741 | 0.026321 | 0.989983 |
| CNN_7 | 0.001955 | 0.04422 | 0.025705 | 0.990215 |
| Metrics  Model | x-coordinate (2 mm/unit) | | | |
|  | MSE | RMSE | MAE | R² |
| CNN_1 | 0.000553 | 0.023523 | 0.018525 | 0.999723 |
| CNN_3 | 0.00004 | 0.006314 | 0.004997 | 0.99998 |
| CNN_5 | 0.000021 | 0.004588 | 0.003631 | 0.999989 |
| CNN_7 | 0.000007 | 0.002654 | 0.00205 | 0.999996 |
| Metrics  Model | y-coordinate (2 mm/unit) | | | |
|  | MSE | RMSE | MAE | R² |
| CNN_1 | 0.000873 | 0.029544 | 0.022877 | 0.999564 |
| CNN_3 | 0.000078 | 0.008838 | 0.006963 | 0.999961 |
| CNN_5 | 0.000017 | 0.004092 | 0.003249 | 0.999992 |
| CNN_7 | 0.000006 | 0.002523 | 0.001991 | 0.999997 |

Supplementary Table S2

**Table S2** Comparison with other works

| **Reference** | **Method** | **Processing model** | **Spatial resolution** | **Force/depth accuracy** | **Fabrication**  **Complexity** | **Computational**  **Load** |
| --- | --- | --- | --- | --- | --- | --- |
| GelSight [24] | Reflective elastomer with markers | CNN | ~2 μm | Normal force 0.67 N | Medium | Medium-High |
| GelSight Svelte [27] | Curved mirrors, flexible backbone | CNN | ~0.03–0.05 mm (varies along sensor) | 9.4 N·mm bend / 7.6 N·mm twist (RMSE) | Medium | High |
| GelTip [23] | Soft elastomer | Numeric method | 5 mm | / | Medium | Low |
| TacTip [18] | Elastomer with markers | Numeric method | 0.20 mm | / | Medium | Medium |
| IrisTact Qiu [26] | Structural color of flexible grating | ResNet | ~0.079 mm | Normal force ~0.006 N | High | High |
| DIGIT [28] | Reflective elastomer, compact design | Struct-NN | sub-millimeters | / | Medium | High |
| TacLINK [17] | Array of markers on soft skin | Finite element model | Mesh 18×9.5 mm | >7 mm depth error | Medium | High |
| ThinTact [32] | Mask-based lensless imaging | Photometric stereo method, Real2Sim | 0.18 mm | 0.13 mm depth error | High | High |
| This work | Microstructure amplification | Ultra-lightweight CNN | MAE<0.04 mm Mesh 2×2 mm | Displacement MAE<0.03mm, normal force <0.005 N | Medium | Low |

Note: “Mesh” indicates the use of structured visual features (e.g., printed marker arrays, checkerboards, or other patterned geometries) for visual deformation tracking in vision-based tactile sensors.

1. M. Shi, Y. Zhang, X. Guo, and E. Yeatman, “Microstructure-Enhanced Vision-Based Tactile Sensor,” in *2023 22nd International Conference on Solid-State Sensors, Actuators and Microsystems (Transducers)*, Kyoto, Japan: IEEE, Jun. 2023. [↑](#footnote-ref-2)
2. M. Shi, Y. Zhang, X. Guo, and E. Yeatman, “Microstructure-Enhanced Vision-Based Tactile Sensor,” in *2023 22nd International Conference on Solid-State Sensors, Actuators and Microsystems (Transducers)*, Kyoto, Japan: IEEE, Jun. 2023. [↑](#footnote-ref-3)
3. Partially created in BioRender. Shi, M. (2026) https://BioRender.com/ll7tk3c [↑](#footnote-ref-4)
